# A family of conserved bacterial virulence factors dampens interferon responses by blocking calcium signaling

**DOI:** 10.1016/j.cell.2022.04.028

**Published:** 2022-06-23

**Authors:** Noémie Alphonse, Joseph J. Wanford, Andrew A. Voak, Jack Gay, Shayla Venkhaya, Owen Burroughs, Sanjana Mathew, Truelian Lee, Sasha L. Evans, Weiting Zhao, Kyle Frowde, Abrar Alrehaili, Ruth E. Dickenson, Mads Munk, Svetlana Panina, Ishraque F. Mahmood, Miriam Llorian, Megan L. Stanifer, Steeve Boulant, Martin W. Berchtold, Julien R.C. Bergeron, Andreas Wack, Cammie F. Lesser, Charlotte Odendall

**Affiliations:** 1 Department of Infectious Diseases, School of Immunology and Microbial Sciences, King’s College London, London, UK; 2 Immunoregulation Laboratory, Francis Crick Institute, London, UK; 3 Center for Bacterial Pathogenesis, Division of Infectious Diseases, Department of Medicine, Massachusetts General Hospital, Boston, MA, USA; 4 Randall Centre for Cell and Molecular Biophysics, King’s College London, London, UK; 5 Department of Biology, University of Copenhagen, Copenhagen, Denmark; 6 Bioinformatics and Biostatistics, The Francis Crick Institute, London, UK; 7 Department of Molecular Genetics and Microbiology, University of Florida College of Medicine, Gainesville, FL, USA; 8 Department of Microbiology, Blavatnik Institute, Harvard Medical School, Boston, MA, USA; 9 Broad Institute of MIT and Harvard, Cambridge, MA, USA

**Keywords:** interferons, *Shigella*, T3SS, OspC1, OspC3, calcium, Ca^2+^, calmodulin, CaMKII, JAK/STAT, ISG, host-pathogen interactions

## Abstract

Interferons (IFNs) induce an antimicrobial state, protecting tissues from infection. Many viruses inhibit IFN signaling, but whether bacterial pathogens evade IFN responses remains unclear. Here, we demonstrate that the *Shigella* OspC family of type-III-secreted effectors blocks IFN signaling independently of its cell death inhibitory activity. Rather, IFN inhibition was mediated by the binding of OspC1 and OspC3 to the Ca^2+^ sensor calmodulin (CaM), blocking CaM kinase II and downstream JAK/STAT signaling. The growth of *Shigella* lacking OspC1 and OspC3 was attenuated in epithelial cells and in a murine model of infection. This phenotype was rescued in both models by the depletion of IFN receptors. OspC homologs conserved in additional pathogens not only bound CaM but also inhibited IFN, suggesting a widespread virulence strategy. These findings reveal a conserved but previously undescribed molecular mechanism of IFN inhibition and demonstrate the critical role of Ca^2+^ and IFN targeting in bacterial pathogenesis.

## Highlights

• Interferons (IFNs) and IFN-stimulated genes (ISGs) restrict *Shigella* infection
• *Shigella* OspC1 and OspC3 effectors block IFN signaling and ISG expression
• OspC effectors bind calmodulin (CaM), inhibiting CaMKII and STAT1 phosphorylation
• Δ*ospC1/C3 Shigella* is attenuated in WT but not in IFN-deficient cells and mice

## Graphical abstract

**Figure:**
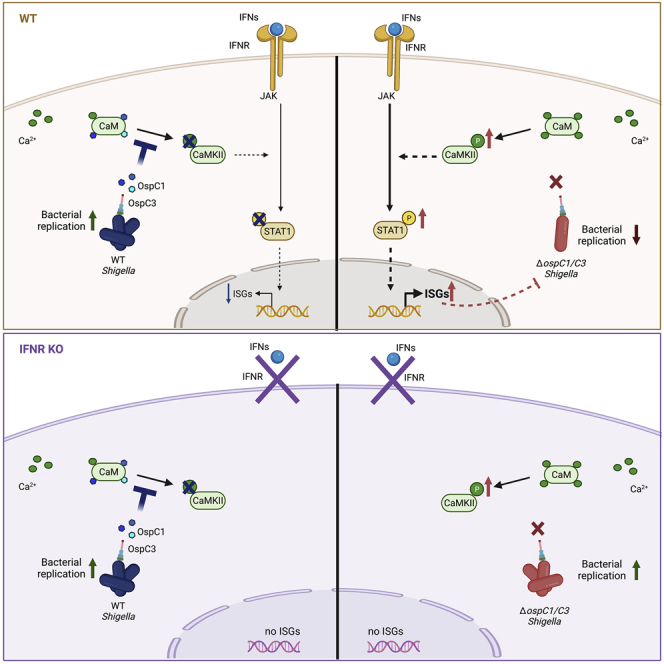

## Teaser

The OspC type-III-secreted effectors in *Shigella* bind calmodulin and block interferon signaling, independently of the cell-death-inhibitory activities of this family of proteins. Conservation of this function in homologs of other bacterial species suggests a common role for Ca^2+^ and interferon targeting in bacterial pathogenesis.

## Introduction

Interferons (IFNs) are families of immunomodulatory cytokines produced in response to infection (De Weerd and Nguyen, 2012). Type II (IFNγ) is the archetypal antibacterial IFN. Type I (IFNαs, β) and type III IFNs (IFNλ1-4) have been historically studied for their antiviral properties, but evidence of their antibacterial roles is emerging (Burke et al., 2020; Helbig et al., 2019; Odendall et al., 2017; Radoshevich et al., 2015; Ranjbar et al., 2015; Snyder et al., 2017). IFNs induce hundreds of interferon-stimulated genes (ISGs), promoting clearance of pathogens and protection from infection. A few ISGs have been established to have direct antibacterial functions (Gaudet et al., 2021; Helbig et al., 2019; Kutsch et al., 2020; Santos et al., 2020; Wandel et al., 2020). For example, human guanylate-binding protein 1 (GBP1) serves as a lipopolysaccharide sensor to activate Caspase 4. This facilitates activation of the noncanonical inflammasome, leading to inflammatory cell death and clearance of intracellular bacteria such as *Shigella* (Kutsch et al., 2020; Santos et al., 2020; Wandel et al., 2020).

*Shigella* spp. are Gram-negative *Enterobacteriaceae*, and the major etiological agents of bacillary dysentery, also called shigellosis (Trofa et al., 1999). Shigellosis, predominantly caused by *Shigella flexneri* and *Shigella sonnei*, is responsible for significant morbidity and mortality worldwide (Anderson et al., 2017; Torraca et al., 2019). Following entry into the gastrointestinal tract, *Shigella* spp invade colonic epithelial cells, rupture their vacuole, and replicate in the cytosol. The pathogenesis of *Shigella* is dependent on its type III secretion system (T3SS), which is encoded on a large virulence plasmid. The T3SS serves as a molecular needle, injecting ∽30 effectors, which are highly conserved among *Shigella* spp., into the host cell to modulate multiple facets of cell physiology and establish a viable niche for infection (Bajunaid et al., 2020; Mattock and Blocker, 2017; Nigro et al., 2019). Among these effectors, the OspC family is emerging as a potent modulator of host immune responses. It comprises the following 3 members: OspC1, OspC2, and OspC3. Despite sharing a high degree of sequence similarity, distinct functions of these effectors have been described. OspC1 was initially shown to promote neutrophil migration (Zurawski et al., 2006). Recent evidence demonstrated that OspC1 also inhibits Caspase 3/7-induced apoptosis, prolonging the survival of infected epithelial cells (Ashida et al., 2020). OspC3 blocks Caspase 4/11-mediated inflammatory cell death (Kobayashi et al., 2013; Li et al., 2021; Mou et al., 2018; Oh et al., 2021), maintaining the bacterium’s epithelial niche during infection.

Viruses are extremely sensitive to IFN-induced responses, and almost all have evolved mechanisms that target components of IFN signaling cascades. Whether bacterial pathogens are capable of the same functions remains unknown. The emerging role of ISGs in the restriction of infection by intracellular bacteria such as *Shigella* led us to hypothesize that *Shigella* might counteract ISG expression. Here, we demonstrate that the conserved family of OspC effectors blocks IFN responses through binding and inhibition of the host Ca^2+^ sensor calmodulin (CaM) (Berchtold and Villalobo, 2014). We show that a *Shigella* strain lacking OspC1 and OspC3 (Δ*ospC1/C3*) strain is attenuated in cell culture and in the murine gut in an IFN-dependent manner, highlighting the importance of IFN inhibition as a virulence strategy. Our data provide a mandate for future work on ISG targeting by bacterial pathogens but also noncanonical IFN inhibition as a common virulence mechanism.

## Results

### *Shigella* effectors block ISG expression

**Figure S1:**
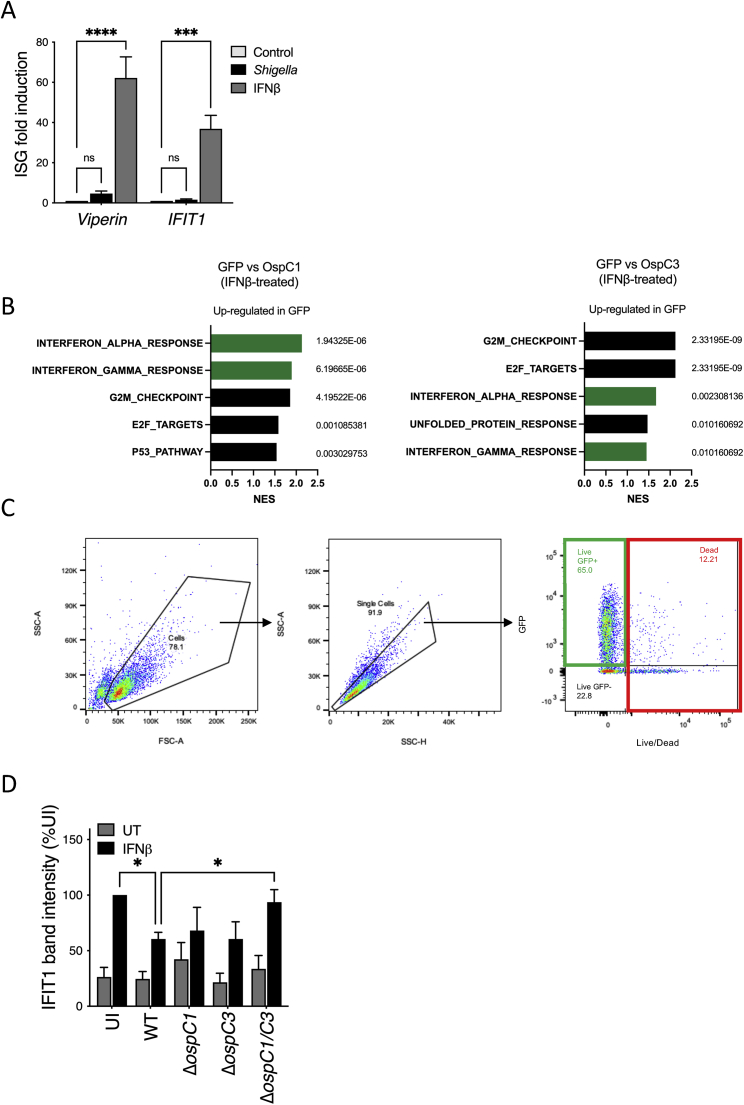
OspC effectors block ISG expression, related to Figure 1 (A) HeLa cells were infected with WT *Shigella sonnei* expressing the adhesin AfaI for 5 h at an MOI of 1. *Viperin* and *IFIT1* mRNA expression was quantified by qRT-PCR. As a comparison, cells were treated with IFNβ (10 ng/mL) for 5 h. (B) RNA-seq analysis of HEK293T cells transfected with cells expressing OspC1, OspC3, and the empty GFP control (GFP), followed by 18 h treatment with IFNβ (10 ng/mL). Normalized enrichment scores (NES) and false discovery rate (FDR) q values (next to bars) for the 5 highest scoring gene sets from the comparison GFP versus OspC1 IFNβ-treated (left) and GFP versus OspC3 IFNβ-treated (right). (C) Gating strategy for Tetherin expression. Debris were eliminated using the forward (FSC) and side scatter (SSC). Within the cell population, single cells were discriminated from doublets. Live cells were discriminated using a live/dead dye (x axis) and transfected cells appeared GFP+ (y axis). Tetherin mean fluorescence intensity (MFI) was calculated within the live GFP+ population (green rectangle). Dead cells shown in red and live GFP− (untransfected cells) were not included in Tetherin MFI measurement. (D) Quantification of IFIT1 western blot shown Figure 1I. Data represent the mean ± SEM of 3 independent experiments. ^∗^ p < 0.05 (t test).

**Figure 1:**
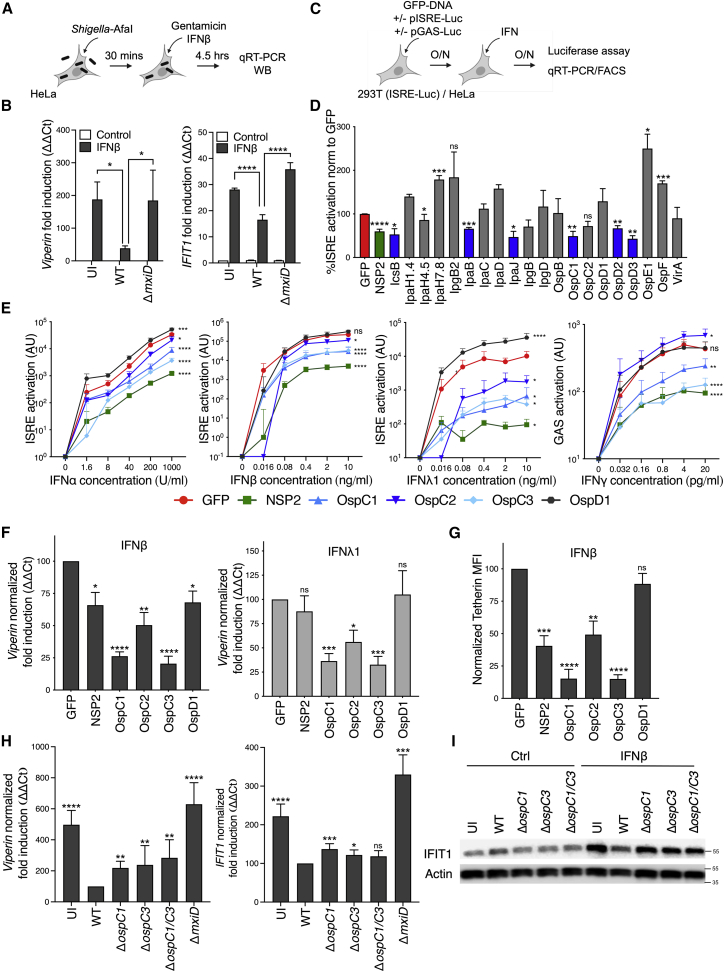
The OspC family of *Shigella* effectors inhibits IFN signaling and ISG expression (A) Experimental layouts for (B), (H), and (I). (B) Uninfected (UI) HeLa cells or cells infected with WT or Δ*mxiD S. sonnei* expressing the adhesin AfaI (multiplicity of infection [MOI] ∼1) were treated with IFNβ for 4.5 h. *Viperin* (left) and *IFIT1* (right) mRNA expression was quantified by qRT-PCR. Data are normalized to untreated control samples. Statistical analysis was performed by two-way ANOVA. (C) Experimental layouts for (D)–(G). (D) HEK293T-ISRE reporter cells transfected with GFP-tagged *Shigella* effector plasmids were treated with IFNβ for 18 h after which luciferase production was monitored. Data are normalized to the empty GFP control vector. Statistical significance was determined by Student’s t test compared with the empty vector control. (E) HEK293T cells cotransfected with GFP-effector expression plasmids plus an ISRE luciferase reporter plasmid were treated for 18 h with a 5-fold dilution series of IFNα, IFNβ, and IFNλ1 (left to right). Alternatively, HeLa cells cotransfected with GFP-effector expression plasmids plus a GAS luciferase reporter plasmid were treated with IFNγ (right panel). Data are expressed as arbitrary luminescence units (AU). Statistical analysis was performed by two-way ANOVA with Dunnett’s post-test, comparing each condition with the empty GFP control. (F) HEK293T cells transfected with GFP-effector expression plasmids were treated with IFNβ (10 ng/mL) or IFNλ1 (20 ng/mL) for 18 h. *Viperin* gene expression was quantified by qRT-PCR, normalized to the empty vector control (GFP). Statistical analysis was performed by one-way ANOVA compared with the empty vector control. (G) HEK293T cells transfected with GFP-tagged effector expression plasmids were treated with IFNβ for 18 h. Tetherin mean fluorescence intensity (MFI) was measured by flow cytometry. Data were normalized to the empty vector control (GFP). Statistical significance was determined by one-way ANOVA. (H) Uninfected (UI) HeLa cells or cells infected with WT, Δ*ospC1*, Δ*ospC3*, or Δ*ospC1/C3 S. sonnei* expressing the adhesin AfaI (MOI ∼ 1) were treated with IFNβ for 4.5 h. *Viperin* and *Ifit1* mRNA expression normalized to WT-infected cells. Statistical analysis was performed using Student’s t test. (I) Similarly to (H), following *Shigella* infection, cells were treated with IFNβ for 4.5 h. IFIT1 protein expression was measured by western immunoblotting. Actin was used as a loading control. Data shown are representative of 4 experiments. qRT-PCR data were calculated using the ΔΔCt relative to the *GAPDH* gene. Data show means ± SEM of 3–6 independent experiments. ns, nonsignificant; ^∗^ p < 0.05; ^∗∗^ p < 0.01; ^∗∗∗^ p < 0.001; ^∗∗∗∗^ p < 0.0001. See also Figure S1.

To identify bacterial strategies that interfere with IFN signaling, we monitored the expression of ISGs in response to *Shigella* infection. We selected HeLa cells for these experiments as they show limited ISG induction in response to infection with *Shigella* but respond robustly to exogenous IFN (Figure S1A). Cells were infected with wild-type (WT) *S. sonnei* or a strain lacking a functional T3SS (Δ*mxiD*). After 30 min, cells were treated with IFNβ for 4.5 h, and ISG expression was quantified by quantitative real-time reverse transcription polymerase chain reaction (qRT-PCR) (Figure 1A). As expected, the ISGs encoding *Viperin* and interferon-induced protein with tetratricopeptide 1 (*IFIT1*) were induced when uninfected (UI) cells were treated with IFNβ (Figure 1B). Infection with WT *Shigella* significantly inhibited ISG expression in response to IFNβ. This inhibition depended on the presence of a functional T3SS, as Δ*mxiD Shigella* infection did not suppress *Viperin* or *IFIT1* expression. This finding implicates T3SS effectors as potential IFN signaling inhibitors. To identify candidate effectors, we utilized a 293T cell line engineered to express luciferase under the control of an interferon-stimulated response elements (ISRE) sequence, which is activated by type I and III IFNs (Odendall and Kagan, 2015; Shapira et al., 2009). Cells expressing individual green fluorescent protein (GFP)-tagged effectors were treated with IFNβ for 18 h (Figure 1C). As a positive control, cells were transfected with a plasmid expressing Chikungunya virus (ChikV) NSP2, a protein known to block IFN signaling (Fros et al., 2010). Expression of several effectors (blue bars) led to IFN inhibition at a level comparable with that mediated by NSP2 (Figure 1D). We chose to follow up on the OspC effector family that had previously been shown to modulate immune pathways (Ashida et al., 2020; Kobayashi et al., 2013; Li et al., 2021; Miller et al., 2018; Mou et al., 2018; Oh et al., 2021; Zurawski et al., 2006).

### The OspC family of *Shigella* effectors inhibits IFN signaling and ISG expression

To determine the spectrum of IFN signaling pathways impacted by OspC family members, we examined the ability of each effector to interfere with type I, II, or III IFN activity. 293T cells cotransfected with each OspC effector and an ISRE reporter were treated with type I IFN (IFNα, IFNβ) or type III IFN (IFNλ1) (Figures 1C and 1E). Given that IFNγ induces a different set of ISGs that contain γ-activated sequences (GAS), IFNγ signaling was assessed following expression of a GAS reporter in HeLa cells (Figures 1C and 1E). ChikV NSP2 was used as a positive control, whereas OspD1, an effector that displayed no inhibition of ISRE in our initial screen (Figure 1D), served as a negative control. We found that OspC1 and OspC3 blocked signaling downstream of increasing concentrations of IFNs from all 3 families (Figure 1E). OspC2 potently inhibited IFNλ1 signaling but was far less efficient at blocking type I IFN signaling and unable to inhibit IFNγ.

We next confirmed that OspC effectors block the expression of endogenous ISGs by monitoring *Viperin* mRNA expression by qRT-PCR in OspC-expressing cells postexposure to IFNβ or IFNλ1 (Figure 1F). Consistent with the reporter analyses described above, OspC1 and OspC3 displayed the most potent ISG suppressive activity. To determine if OspCs globally suppressed ISG expression, we employed RNA-seq analysis of cells expressing the empty GFP control, OspC1 or OspC3, treated or not with IFNβ. We performed gene set enrichment analysis (GSEA) comparing IFNβ-treated GFP with OspC1- or OspC3-expressing cells to identify the hallmark pathways most affected by the presence of OspC family members. In both comparisons, IFN response pathways were among the top five pathways enriched in control samples (GFP) (Figure S1B), indicating global suppression of IFN response pathways by OspC. Finally, we tested whether ISG expression was blocked at the protein level by quantifying cell surface expression of the ISG Tetherin by flow cytometry. After gating on live transfected cells (Figure S1C), we found that Tetherin expression was blocked in cells expressing OspC1 and OspC3 (Figure 1G).

Next, we sought to determine if OspC family members translocated at endogenous levels by *Shigella* during infection impact IFN signaling. HeLa cells infected with WT *Shigella* or strains lacking either *ospC1*, *ospC3*, or both (Δ*ospC1/C3*) were treated with IFNβ, and ISG expression was assessed. As previously observed, infection with WT bacteria inhibited expression of *Viperin* and *IFIT1* (Figure 1H). Infection with Δ*ospC1*, Δ*ospC3*, or Δ*ospC1/C3* resulted in higher levels of IFNβ-mediated ISG expression than infection with WT bacteria (Figure 1H). Analysis of protein levels by western blot demonstrated that IFIT1 expression was also significantly diminished when cells infected with WT *Shigella*, but not strains lacking *ospC1* and/or *ospC3*, were exposed to IFNβ (Figure 1I and quantification Figure S1D). These data indicate that OspC1 and OspC3 contribute to the inhibition of IFN signaling in epithelial cells infected with *Shigella*. However, when compared with Δ*mxiD*-infected cells, the rescue we observed in Δ*ospC1/C3*-infected cells was only partial, likely suggesting a contribution from other effectors identified in our screen (Figure 1D).

### Inhibition of IFN is distinct from inhibition of cell death by OspC effectors

**Figure S2:**
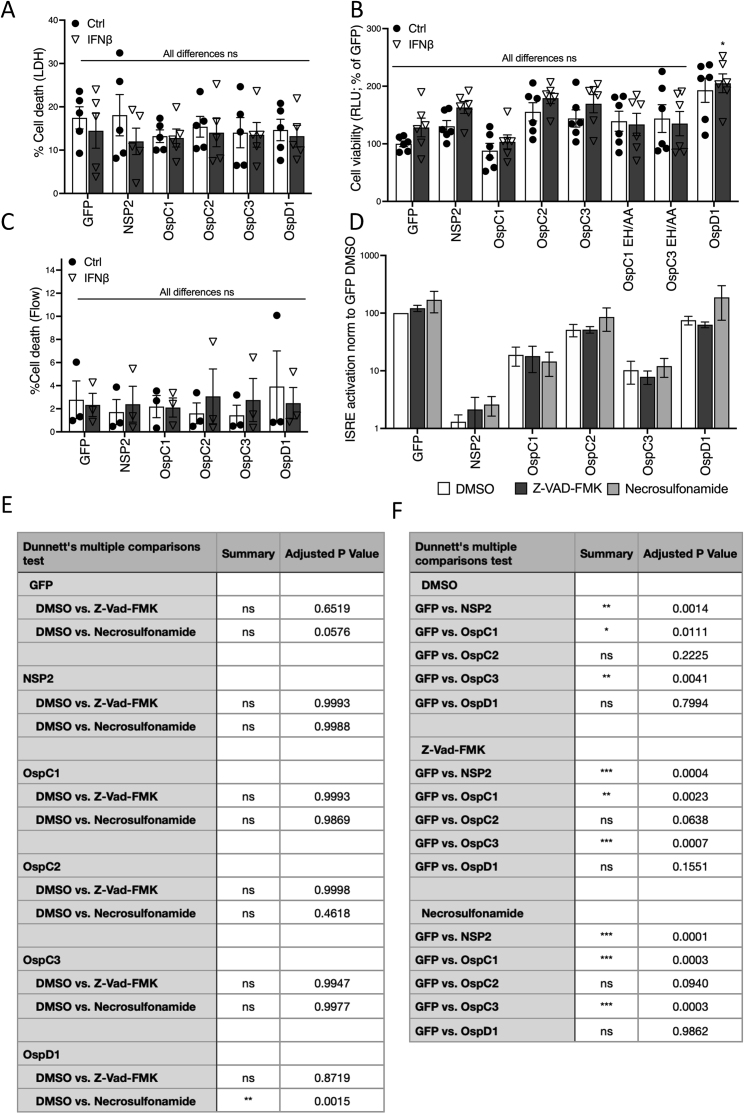
OspC 1 and 3 inhibition of IFN signaling is independent of Caspase-mediated cell death, related to Figure 2 (A) LDH release in HEK293T cells transfected with GFP-tagged effector plasmids followed by 18 h treatment or not with IFNβ (10 ng/mL). Data are expressed as percentage of LDH release relative to a lysis control. Data represent the mean ± SEM of five independent experiments. (B) Similar to (A) except cell viability was measured from intracellular ATP concentrations using Cell Titer Glo. Data are expressed as a percentage of the values in GFP transfected, unstimulated (Ctrl) cells. Data represent the mean ± SD of 2 independent experiments. (C) Similar to (A) except cell viability was measured by flow cytometry with a live/dead stain (see Figure S1B for gating strategy). Data represent the mean ± SEM of three independent experiments. (A–C) Statistical analyses were carried out using two-way ANOVA with Tukey’s multiple comparisons test. (D) HEK293T cells co-transfected with GFP-tagged effector plasmids and an ISRE reporter were treated with cell death inhibitors Z-VAD-FMK (25 μM) or Necrosulfonamide (20 μM) before IFNβ (10 ng/mL) stimulation for 18 h. Data show means ± SEM of at least three independent experiments. (E and F) Statistical comparison of data presented in (B). Analyses of DMSO versus drug treated sample (E) or GFP versus other constructs (F) were conducted using one-way ANOVA with Dunnett’s multiple comparisons test. ns, nonsignificant; ^∗^ p < 0.05; ^∗∗^ p < 0.01; ^∗∗∗^ p < 0.001.

OspC1 and OspC3 have previously been reported to inhibit cell death pathways. OspC1 targets an unknown host factor to inhibit Caspase 3/7-mediated apoptosis (Ashida et al., 2020), whereas OspC3 binds Caspase 4/11 to inhibit pyroptosis via its ADP-riboxanase activity (Kobayashi et al., 2013; Li et al., 2021; Oh et al., 2021). We next investigated whether inhibition of IFN signaling by OspC1 and OspC3 was related to their cell death inhibition. Expression of OspC effectors in 293T cells in the absence or presence of IFNβ had no effect on host cell viability, as assessed by quantifying released lactate dehydrogenase (LDH) (Figure S2A), intracellular ATP—a marker of active metabolism (Figure S2B) or by using flow cytometry to determine the percentages of live versus dead cells (Figures S2C and S1C). In addition, inhibition of pyroptosis and apoptosis with Z-Val-Ala-Asp-fluoromethylketon (Z-VAD-FMK) or necroptosis with necrosulfonamide had no effect on ISRE activation (Figures S2D and S2E). Finally, the inhibitory activities of OspC1 and OspC3 were identical in the presence of a DMSO vehicle control or cell death inhibitors (Figures S2D and S2F). These data demonstrate that expression of OspC1 or OspC3 in combination with IFN treatment is not cytotoxic. In addition, we show that pharmacological inhibition of pyroptosis, apoptosis, or necroptosis neither phenocopy OspC1 or OspC3 expression in blocking IFN signaling nor affect their ability to block IFN signaling.

**Figure S3:**
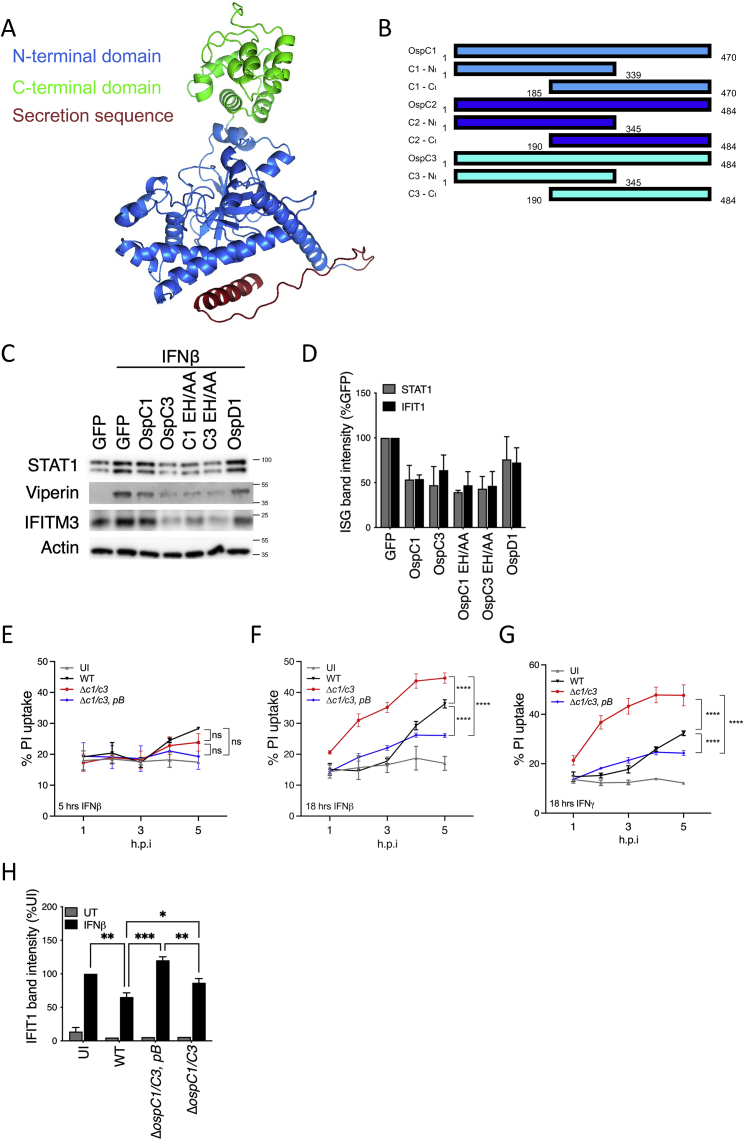
N-terminal fragments of OspC 1, and OspC3 inhibit ISG expression, related to Figure 2 (A) 3D structure of OspC3 derived from AlphaFold. The N-terminal domain is depicted in blue and the C-terminal domain in green. The predicted secretion sequence is depicted in red. (B) Diagrammatic representation of OspC truncation vectors. OspC1 truncations are shown is blue, OspC2 in royal blue and OspC3 in turquoise. C- and N-terminal amino acid positions are indicated for each construct. (C) Mouse Swiss3T3 cells were transfected with OspC1, OspC3 or their associated E326A and H328A (EH/AA) point mutants and treated with murine IFNβ (1,000 U/mL) overnight. STAT1, IFIT1, and IFITM3 protein expression was measured by western immunoblotting. Actin was used as a loading control. Data shown are representative of 2 independent experiments. (D) Densitometry quantification of experiment shown Figure 2E. Data represent the mean ± SEM of 3 independent experiments. Statistical analysis was performed with two-way ANOVA. (E) Uninfected (UI) HeLa cells or cells infected with *S. sonnei* (WT), *ΔospC1/C3 Shigella*, or *ΔospC1/C3, pB Shigella* expressing the adhesin AfaI at a MOI of 10. 30 min postinvasion, cells were treated with IFNβ (10 ng/mL) for 4.5 h. PI uptake was monitored at the indicated time points. Data are expressed as the percentage of PI uptake compared with wells treated with Triton-X100. Statistical analysis between PI uptake profiles was performed with a two-way ANOVA with Dunnett’s multiple comparisons. Data are representative of 2 independent experiments (Error bars represent SD). (F and G) Similarly to (E), cells were treated with IFNβ (10 ng/mL, F) or IFNγ (10 ng/mL, G) for 18 h prior to infection, and infected at an MOI of 50. (H) Quantification of IFIT1 immunoblot shown in Figure 2G. Data represent the mean ± SEM of 3 independent experiments. Statistical analysis between was performed with one-way ANOVA. ^∗^ p < 0.05; ^∗∗^ p < 0.01; ^∗∗∗^ p < 0.001; ^∗∗∗∗^ p < 0.001.

**Figure 2:**
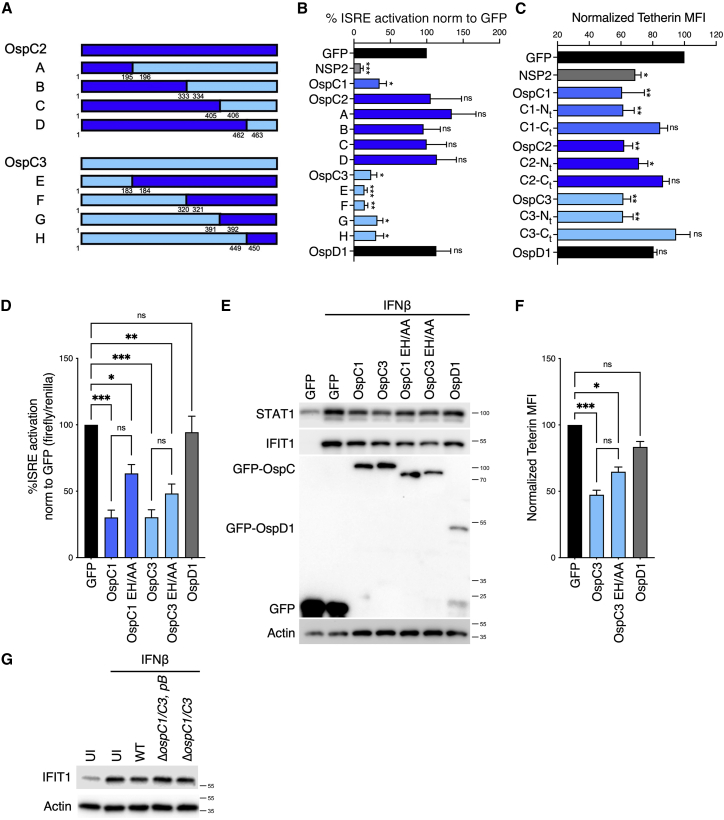
OspC effectors inhibit IFN signaling and cell death via distinct molecular mechanisms (A) Diagrammatic representation of chimeric GFP-tagged OspC2/3 effectors. OspC2 fragments are shown in royal blue, whereas OspC3 are shown in turquoise. (B) HEK293T cells were cotransfected with an ISRE luciferase reporter and GFP-tagged chimeric effector plasmids. Cells were treated for 18 h with IFNβ. Statistical analysis was carried out using one-way ANOVA. (C) HEK293T cells transfected with GFP-tagged full-length OspC plasmids or N- or C-terminal truncation mutants of OspC effectors (shown diagrammatically in supplementary Figure S3B) were treated for 18 h with IFNβ. Tetherin mean fluorescence intensity (MFI) was measured by flow cytometry. Statistical analysis was performed by one-way ANOVA with Dunnett’s post-test, comparing each condition with the empty GFP control. (D) Similarly to (B), except OspC1 and OspC3, E326A and H328A (EH/AA) point mutants were used in addition to wild-type plasmids. To control for cell viability, cells were cotransfected with a Renilla luciferase internal control vector. Statistical analysis significance was determined by one-way ANOVA. (E) Cell lysates from experiment carried out in (D) were subjected to SDS-PAGE. STAT1 and IFIT1 protein expression was measured by western immunoblotting. GFP was used as control of transfection efficiency. (F) Similarly to (C), except that GFP-tagged OspC3, OspC3 EH/AA, or OspD1 expression plasmids were used. Statistical significance was determined by one-way ANOVA. (G) Uninfected (UI) HeLa cells or cells infected with WT, Δ*ospC1/C3* or Δ*ospC1/C3* expressing an OspC2/OspC3 chimera (Δ*ospC1/C3*, pB− B in Figure 2A) *S. sonnei* expressing the adhesin AfaI (MOI ∼ 10) were treated with IFNβ for 4.5 h. IFIT1 protein expression was visualized by immunoblotting. (B–D and F). Data show means ± SEM of 3 independent experiments. (E and G) For immunoblotting, actin was used as a loading control, and data shown are representative of at least 3 experiments. ns, nonsignificant; ^∗^ p < 0.05; ^∗∗^ p < 0.01; ^∗∗∗^ p < 0.001. See also Figures S2 and S3.

Our prediction of the OspC3 tertiary structure using AlphaFold (Jumper et al., 2021) suggests the presence of distinct N- and C-terminal domains (Figure S3A). The C terminus of OspC3 is required for binding to Caspase 4/11 (Kobayashi et al., 2013; Li et al., 2021). We reasoned that if cell death and IFN signaling inhibition are independent activities, distinct regions within OspC effectors may be required for these activities. By swapping N- and C-terminal regions of OspC3 and OspC2 (Figure 2A), the latter of which has limited IFNβ inhibitory activity in our reporter assay (Figure 1E), we mapped the region of OspC3 that is sufficient to prevent IFN signaling (Figure 2B). We found that chimeras A-D, which contain regions of the OspC2 N terminus and the OspC3 C terminus (shown in royal blue in 2B), were not able to block IFNβ signaling in our reporter assay. By contrast, chimeras E-H (shown in turquoise in 2B) that contained the N-terminal portion of OspC3 potently inhibited IFN signaling. Thus, the N-terminal region of OspC3 within amino acids (aa) 1–183 (as in chimera E) is required to restrict IFNβ activity.

To confirm these observations, we constructed 2 sets of truncation mutants. The first set (denoted N_t_) contains the entire N-terminal domain of OspC1, OspC2, or OspC3 (Figure S3B), but lack the Caspase 4-binding region, shown to be essential for pyroptosis inhibition (Ashida et al., 2020; Li et al., 2021). The second set (denoted C_t_) contains most of each OspC protein, including the entire C-terminal domain, lacking only the putative IFN inhibitory motif identified in the chimera experiments (Figures 2A, 2B, and S3B). Only the N-terminal fragments of each OspC effector were able to block Tetherin expression following IFNβ treatment (Figure 2C).

The ability of OspC3 to block pyroptosis was recently demonstrated to be attributed to its ADP-riboxanation of Caspase 4, an enzymatic activity shared by other OspC effectors (Li et al., 2021). In their study, Li and colleagues showed that this activity is dependent on several residues, including E326 and H328. Mutation of E326 and H328 to alanines (EH/AA) abrogates ADP-riboxanase activity, preventing OspC3 from modifying Caspase 4 and inhibiting pyroptosis (Li et al., 2021). We generated OspC1 and OspC3 alleles that harbor these EH/AA mutations. These enzymatically inactive proteins retained their ability to block the activation of ISRE (Figure 2D), as well as the induction of ISGs, as assessed by western immunoblotting against endogenous proteins in human (Figure 2E, quantification Figure S3D) and mouse cells (Figure S3C). Finally, OspC3 EH/AA also blocked IFNβ-induced Tetherin expression (Figure 2F).

**Figure 3:**
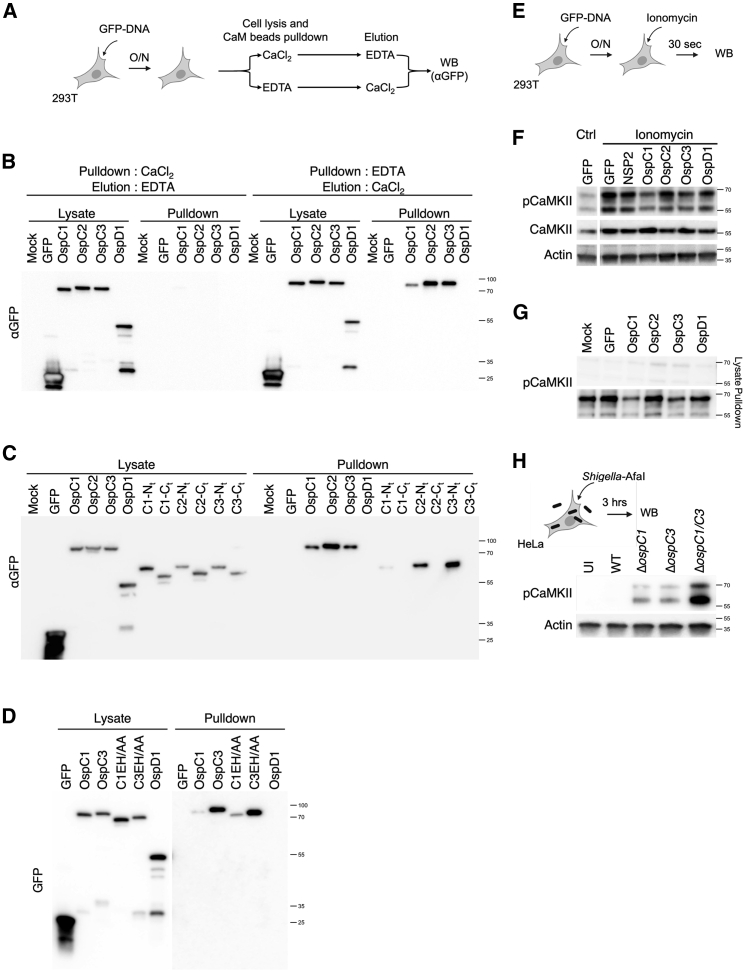
OspC1 and OspC3 bind and inhibit calmodulin (CaM) (A) Experimental layout for (B–D). (B) Lysates of HEK293T cells expressing GFP-tagged effectors were incubated with calmodulin (CaM) beads. Cell lysis and pulldown were carried out in the presence of buffer containing CaCl_2_ or EDTA to determine interaction with Ca^2+^-bound or Ca^2+^-free CaM, respectively. Bound proteins were eluted using the opposite condition. The presence of GFP-tagged proteins in the lysates or pulldown was analyzed by western immunoblotting. (C) Similar to (B), except N- or C-terminal truncation mutants of OspC effectors were studied (shown in Figure S3B). Pulldown was conducted in buffer containing EDTA, whereas elution was performed using CaCl_2_. (D) Similar to (C), except OspC1 and OspC3, E326A and H328A (EH/AA) point mutants were studied. (E) Experimental layout for (F). (F) HEK293T cells expressing GFP-tagged effectors were treated with 2 μM ionomycin for 30 s. CaMKII phosphorylation was analyzed by immunoblotting. (G) Similar to (B), lysates of cells expressing GFP-effectors were subjected to affinity purification with CaM beads in the presence of CaCl_2_. Interaction with pCaMKII was analyzed by immunoblotting. (H) HeLa cells were infected with the indicated *S. sonnei* strains expressing the adhesin AfaI (MOI ∼ 1) for 3 h. CaMKII phosphorylation was analyzed by immunoblotting. For immunoblotting, actin and/or total proteins were used as loading controls. Data shown are representative of 3–5 experiments. See also Figures S4 and S5.

Next, we introduced chimera B (Figure 2A) into Δ*ospC1/C3 Shigella*. This chimera contains the OspC3 Caspase 4 binding site and all residues necessary for its ADP-riboxanation, but not the residues necessary for IFN inhibition (Figure 2B). As predicted, infection with the resulting strain Δ*ospC1/C3*, *pB* led to minimal cell death, when compared with Δ*ospC1/C3 Shigella* after IFNβ or IFNγ treatment (Figures S3E–S3G). Importantly, this strain blocked cell death at least as efficiently as WT *Shigella* but was incapable of blocking IFNβ-mediated IFIT1 expression (Figure 2G, quantification Figure S3H).

Taken together, these results demonstrate that the C-terminal domain and ADP-riboxanase activity of OspC3, which are essential for Caspase 4 inhibition, are dispensable for its inhibition of IFN signaling and ISG expression. Therefore, these findings establish that OspC effectors inhibit cell death and IFN signaling by distinct biochemical mechanisms.

### OspC1 and OspC3 bind and inhibit calmodulin (CaM)

**Figure S4:**
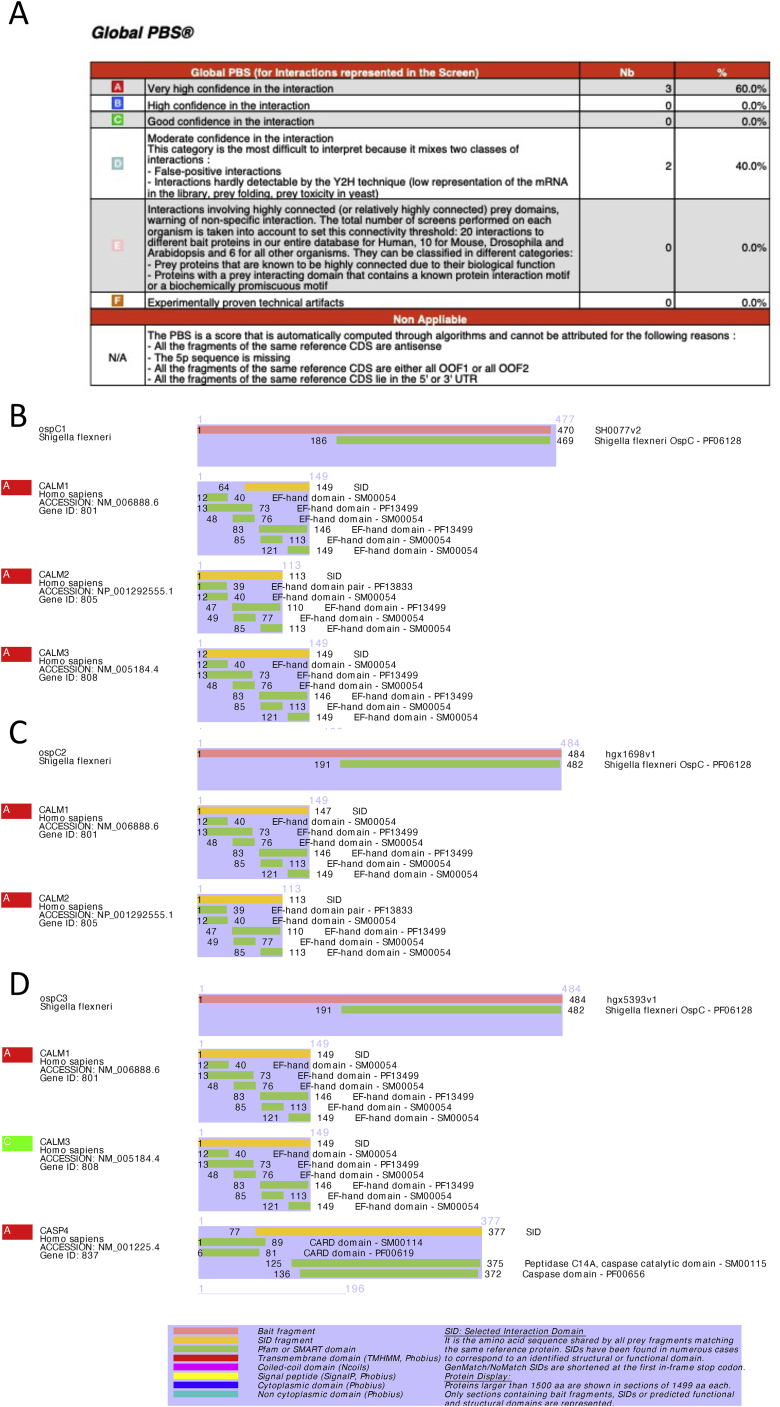
OspC1, OspC2, and OspC3 interact with calmodulin, related to Figure 3 (A) Legend for interpretation of Figures S4B–S4D. (B–D) OspC1, 2, and 3 underwent a yeast 2-hybrid interaction assay. High-confidence interactor partners are shown for OpsC1 (B), OspC2 (C), and OspC3 (D).

In an effort to identify additional candidate host binding partners of OspC effectors, full-length OspC1, OspC2, and OspC3 were each used as bait in a yeast two-hybrid screen with a cDNA fragment library generated from human macrophages activated with the Toll-like receptor 2 ligand Pam3CSK4 and IFNβ. Candidate interacting proteins were assigned a predicted biological score of A to F (Formstecher et al., 2005), with A corresponding to a very high confidence in the interaction (Figure S4A). Between 60 and 100 million interactions were tested for each effector. For all three OspC effectors, over 150 clones were obtained, the vast majority of which contained a prey fragment encoding CALM (calmodulin, CaM) with a score of A (Figures S4B–S4D). As predicted (Kobayashi et al., 2013), and as a validation of our screen, OspC3 was found to interact with Caspase 4 (Figure S4D).

CaM is a 17 kDa protein and a host Ca^2+^ sensor (Berchtold and Villalobo, 2014). When bound to Ca^2+^, CaM adopts an open conformation facilitating its binding to, and activation of, a large number of downstream targets including calmodulin kinase II (CaMKII). We determined whether OspC effectors interacted with Ca^2+^-bound or Ca^2+^-free (Apo−) CaM. Lysates of 293T cells expressing GFP-tagged proteins of interest were incubated with CaM-sepharose beads in buffer containing either CaCl_2_ or the Ca^2+^ chelator ethylenediaminetetraacetic acid (EDTA), followed by elution in the opposite condition (Figure 3A). We observed no interactions in Ca^2+^-enriched conditions (Figure 3B, left half of blot). However, all three OspC effectors interacted with Apo-CaM (Figure 3B, right half of blot). Importantly, we detected interaction with the N-, but not the C-terminal fragments of each effector (Figure 3C). Additionally, OspC1 and OspC3 EH/AA mutants bound Apo-CaM as efficiently as their WT counterparts (Figure 3D). These data, along with our findings in Figures 2B–2G, demonstrate that interaction with CaM and inhibition of IFN signaling occur via the N termini of OspC1 and OspC3 and independently of their cell death inhibitory activities.

**Figure S5:**
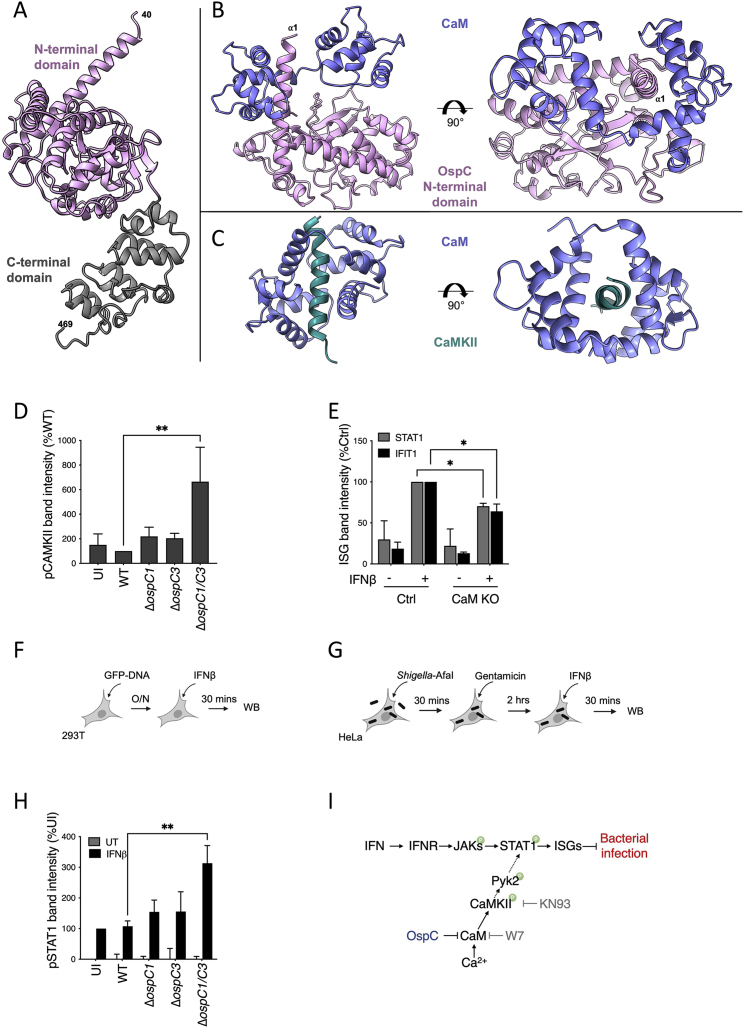
OspC effectors block STAT1 phosphorylation, related to Figures 3 and 4 (A) Structural model of OspC1, predicted using AlphaFold with the N-terminal domain (40–337) in pink and the C-terminal domain (338–469) in gray. The location of the first helix (⍺1) is indicated. (B) AlphaFold-generated model of calmodulin (CaM) bound to OspC1. The two lobes of CaM (purple) are wrapped around the ⍺1 helix of OspC1. (C) Crystal structure of the CaM-CaMKII complex (PDB: 2WEL). As for OspC1, the two lobes of CaM (purple) wrap around a single N-terminal helix of CaMKII (teal). (D) Quantification of phosphorylated CaMKII immunoblot shown Figure 3H. (E) Quantification of IFIT1 and STAT1 immunoblots shown Figure 4D. (F) Experimental layout for Figure 4H. (G) Experimental layout for Figure 4I. (H) Quantification of phosphorylated STAT1 immunoblot shown in Figure 4I. Data represent the mean ± SEM of 5 (D), 3 (E and H) independent experiments. Statistical analyses were carried out using one- (D) or two-way ANOVA (E and H). ^∗^ p < 0.05; ^∗∗^ p < 0.01. (I) Diagram summarizing our findings: IFNs restrict *Shigella* infection via the JAK/STAT-mediated induction of ISGs. Ca^2+^/CaM/CaMKII modulate IFN signaling by potentiating STAT1 phosphorylation. *Shigella* OspC1 and OspC3 bind and inhibit CaM to block CaMKII phosphorylation, STAT1 activation and ISG expression.

We next used AlphaFold to model the OspC1-CaM complex. This led to a high-confidence model, with the two lobes of CaM wrapped around an α helix protruding from the OspC1 N-terminal domain (Figures S5A and S5B). This interaction is reminiscent of CaM binding to CaMKII, whereby the two lobes of CaM are similarly wrapped around a helix at the N terminus of CaMKII (Figure S5C). This suggests a model whereby OspC recruits and sequesters CaM in the absence of Ca^2+^, thus preventing its downstream activation of CaMKII.

We hypothesized that targeting of Apo-CaM by OspC1 and OspC3 might prevent CaM from binding and activating its signaling partners. When bound to Ca^2+^, CaM binds and activates CaMKII by inducing its autophosphorylation on T286. Cells expressing OspC effectors were treated with the Ca^2+^ ionophore ionomycin for 30 s to increase CaMKII phosphorylation (Figures 3E and 3F). T286 phosphorylation was reduced upon expression of OspC1 or OspC3 but not OspC2 (Figure 3F). Importantly, ChikV NSP2, which blocks IFN signaling via a different mechanism (Fros et al., 2010), had no effect on CaMKII activation. pCaMKII binding to CaM was similarly inhibited by OspC1 or OspC3 but not OspC2 (Figure 3G). Moreover, although HeLa cells infected with WT *Shigella* exhibited little to no CaMKII phosphorylation, those infected with strains lacking either OspC1 or OspC3 demonstrated evidence of T286 phosphorylation that was further increased within cells infected with Δ*ospC1/C3 Shigella* (Figure 3H, quantification Figure S5D). These data therefore establish that OspC1 and OspC3 synergize to block CaMKII activation.

### CaM/CaMKII modulate JAK/STAT and IFN signaling

**Figure 4:**
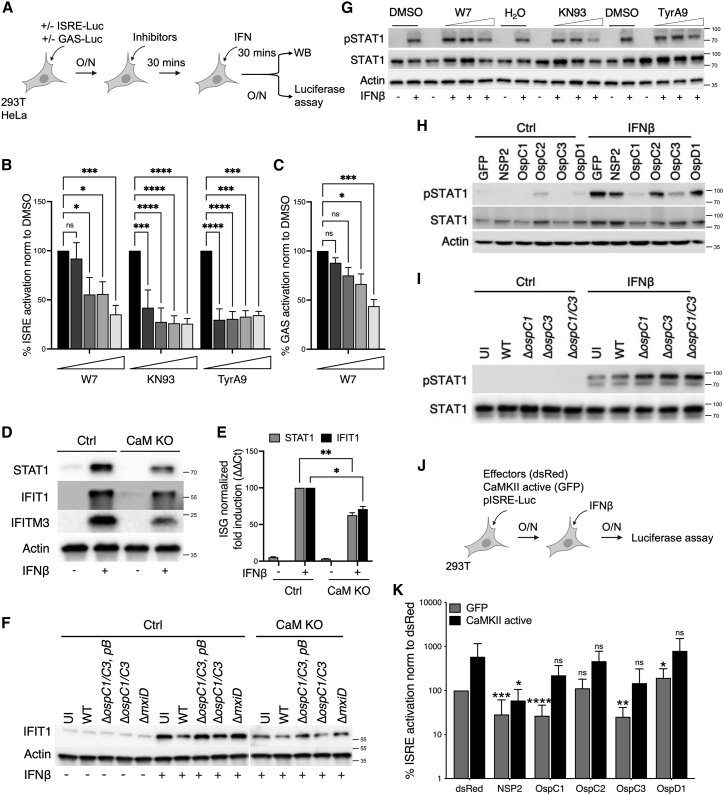
CaM/CaMKII modulate JAK/STAT and IFN signaling (A) Experimental layouts for (B), (C), and (G). (B) HEK293T cells were cotransfected with ISRE firefly and Renilla reporters. Cells were treated with 2-fold dilutions of W7, KN93, or TyrA9 30 min prior to the addition of IFNβ for 18 h. Statistical analyses were performed by one-way ANOVA comparing drug exposed samples with their no drug controls. (C) Similar to (B), except HeLa cells expressing a GAS reporter were treated with W7 and IFNγ. (D) Tet-OFF parental HeLa cells (Ctrl) or cells depleted for CaM (CaM KO) were treated with 1 μg/mL doxycycline for 72 h, before treatment with IFNβ for 18 h. STAT1, IFIT1, and IFITM3 protein expressions were analyzed by immunoblotting. (E) Similar to (D), except *STAT1* and *IFIT1*, mRNA expressions were quantified by qRT-PCR. Statistical significance was determined by two-way ANOVA. (F) Ctrl or CaM KO HeLa cells were treated with doxycycline as in (D). Uninfected (UI) cells or cells infected with WT, Δ*ospC1/C3* or Δ*ospC1/C3* expressing an OspC2/OspC3 chimera (Δ*ospC1/C3*, pB−B in Figure 2A) *S. sonnei* expressing the adhesin AfaI (MOI ∼ 10) were treated with IFNβ for 4.5 h. IFIT1 protein expression was visualized by immunoblotting. (G) Similar to (B), except STAT1, phosphorylation was analyzed in HEK293T cells treated with IFNβ for 30 min. (H) HEK293T cells were transfected with the indicated effectors and treated with IFNβ for 30 min (experimental layout shown in Figure S5F). STAT1 phosphorylation was analyzed by western immunoblotting. (I) HeLa cells were infected with the indicated AfaI-expressing *Shigella* strains (MOI∼ 1) for 3 h, followed by 30 min treatment with IFNβ. STAT1 phosphorylation was analyzed by western immunoblotting (experimental layout shown in Figure S5G). (J) Experimental layout for (K). (K) HEK293T cells were cotransfected with an ISRE luminescence reporter, dsRed-tagged effector plasmids and either empty GFP, or GFP-tagged active CaMKII (T286D, T305/6A), followed by treatment with IFNβ for 18 h. Luminescence data were normalized to the empty dsRed, empty GFP control. Statistics represent Student’s t test comparing each dsRed effector with the empty control, in either GFP or CaMKII active expressing cells. Data show means ± SEM of 3–5 independent experiments. For immunoblotting, actin and/or total proteins were used as loading controls. Data shown are representative of 3–5 experiments. ns, nonsignificant; ^∗^ p < 0.05; ^∗∗^ p < 0.01; ^∗∗∗^ p < 0.001; ^∗∗∗∗^ p < 0.0001. See also Figure S5.

IFNα or IFNγ signaling pathways were shown to be modulated by CaM and CaMKII in macrophages, but whether this is the case for IFNβ in epithelial cells had not been studied (Nair et al., 2002; Wang et al., 2008). Our findings that N-terminal fragments of OspC1 and OspC3 both bind CaM and inhibit IFN suggested that these two phenotypes are linked. Thus, we investigated whether pharmacological inhibition or genetic depletion of CaM would recapitulate the effects of OspC1 and OspC3 expression and block IFN signaling. First, 293T cells expressing an ISRE reporter were treated with inhibitors of CaM (W7), CaMKII (KN93), and its downstream signaling partner Pyk2 (Tyrphostin A9-TyrA9) (Broeke et al., 2004; Lv et al., 2021; Wang et al., 2009), followed by stimulation with IFNβ (Figures 4A and 4B). In parallel, HeLa cells expressing a GAS reporter were treated with W7 and stimulated with IFNγ (Figures 4A and 4C). W7 blocked ISRE and GAS activation in a dose-dependent manner (Figures 4B and 4C). Inhibition of CaMKII with KN93 or Pyk2 with TyrA9 blocked IFNβ signaling (Figure 4B). Second, genetic depletion of CaM (Munk et al., 2020) reduced the ability of HeLa cells to induce ISGs following IFNβ treatment as assessed by immunoblotting for STAT1, IFIT1, or IFITM3 (Figure 4D, quantification Figure S5E) and by monitoring the levels of ISG mRNAs via qRT-PCR (Figure 4E). Finally, the increase we observed in IFNβ-mediated IFIT1 expression following Δ*ospC1/C3* or Δ*ospC1/C3, pB* infection was ablated in cells depleted for CaM (Figure 4F).

All IFNs signal via the Janus kinase (JAK)-mediated phosphorylation of signal transducer and activator of transcription 1 (STAT1). As IFN-mediated STAT1 phosphorylation was shown to be blocked by inhibition of CaM signaling in primed macrophages (Nair et al., 2002; Wang et al., 2008), we investigated whether CaM/CaMKII contributes to STAT1 phosphorylation in our experimental system (Figures 4A and 4G). W7, KN93, and TyrA9 all inhibited STAT1 phosphorylation. These data suggest that the CaM/CaMKII pathway modulates ISRE and ISG expression via phosphorylation of STAT1.

We therefore hypothesized that if OspC1 and OspC3 inhibited ISG expression by targeting CaM, they should block STAT1 phosphorylation. Expression of OspC1 and OspC3 completely abolished STAT1 phosphorylation in response to IFNβ treatment (Figures 4H and S5F). We also observed elevated IFNβ-induced STAT1 phosphorylation in HeLa cells infected with strains lacking *ospC1*, *ospC3*, or both (Figures 4I and S5G, quantification Figure S5H).

Finally, we reasoned that if OspC1 and OspC3 block IFN signaling by preventing CaM from activating CaMKII, overexpression of active CaMKII along with these effectors might bypass their ability to block ISG expression. Thus, GFP- or GFP-tagged constitutively active CaMKII (T286D, T305/6A) (Chang et al., 2019) expression plasmids were cotransfected with dsRed-OspC effector plasmids and our ISRE luciferase reporter (Figures 4J and 4K). Expression of active CaMKII increased IFN-mediated activation of ISRE by almost 10-fold, compared with GFP (compare gray and black bars in dsRed conditions; Figure 4K). These data show that constitutively active CaMKII increases IFN signaling. We next tested whether active CaMKII can bypass the inhibitory effects of OspC1 and OspC3. When coexpressed with GFP, dsRed-tagged OspC1 or OspC3 inhibited IFNβ-mediated activation of ISRE 5-fold, whereas OspC2 was not inhibitory (Figure 4K, gray bars). However, in the presence of active GFP-CaMKII, OspC1 and OspC3 were no longer able to significantly block ISRE activation (Figure 4K, black bars). This is in contrast with NSP2 that blocked IFN signaling almost equally in the presence or absence of active CaMKII. Taken together, these data demonstrate that OspC1 and OspC3 target the CaM/CaMKII pathway to block STAT1 phosphorylation and ISG expression (Figure S5I).

### OspC effectors are phylogenetically and functionally conserved across multiple bacterial pathogens

**Figure 5:**
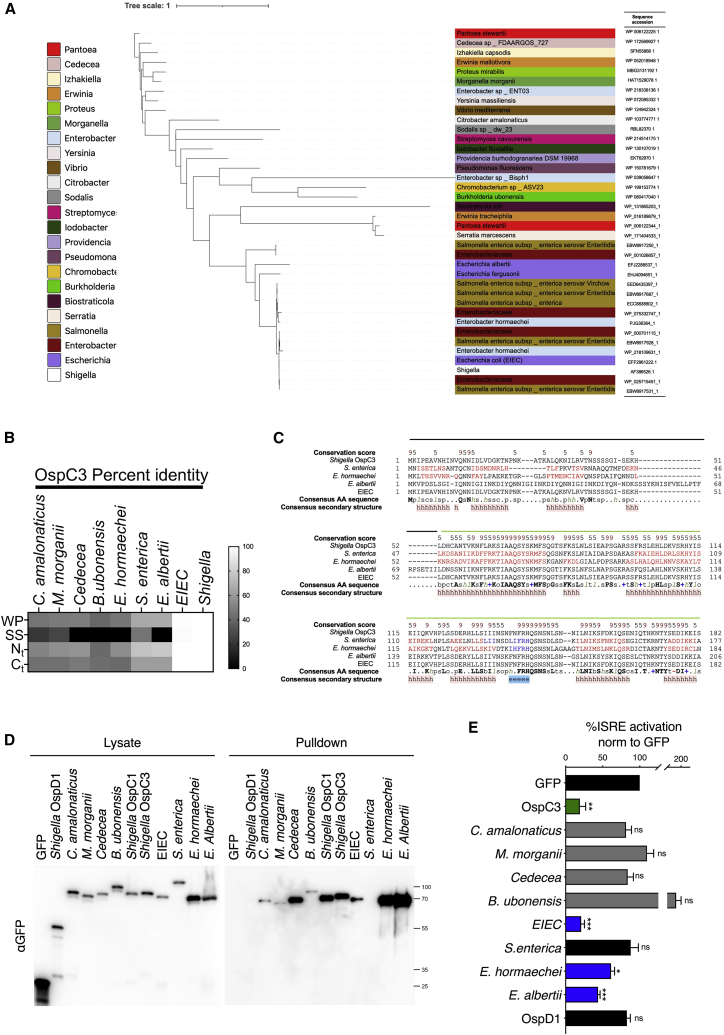
OspC effectors are phylogenetically and functionally conserved across multiple bacterial pathogens (A) Maximum likelihood phylogenetic tree showing the relatedness between *Shigella* OspC3 and its closest homologs in the NCBI database generated in iTol. (B) Amino acid identity matrix comparing distinct regions of the OspC protein with 8 homologs. WP, whole protein; SS, disordered region/predicted secretion sequence; N_t_, N-terminal domain; C_t_, C-terminal domain. (C) Representative sequence alignment of *Shigella* OspC3 and 4 representative homologs from amino acid 1 to 252. Predicted secondary structures are displayed below the alignment, and conservation scores are shown above the alignment. The predicted secretion sequence is indicated by a black bar, whereas the N-terminal domain is indicated in green. h, helix; e, extended strand of β sheet. (D) GFP-tagged representative OspC homologs were subjected to affinity purification with CaM beads. Cell lysis and pulldown were carried out in buffer containing EDTA, before elution in CaCl_2_ (experimental layout shown in Figure 3A). Data shown are representative of 4 experiments. *E. albertii* pulldown samples produced consistently higher chemiluminescent signal compared with other samples in the same experiment. The gel therefore represents *E. albertii* from a different experimental replicate, from the other displayed samples. (E) HEK293T cells cotransfected with GFP-effector expression plasmids plus an ISRE luciferase reporter plasmid were treated for 18 h with IFNβ before subjecting lysates to luciferase assay. Data are expressed as percentage of the empty GFP control vector. Data show means ± SEM of 4 independent experiments. Statistical analyses were performed by Student’s t test compared with the empty GFP control. ^∗^ p < 0.05; ^∗∗^ p < 0.01; ^∗∗∗^ p < 0.001. See also Figure S6.

**Figure S6:**
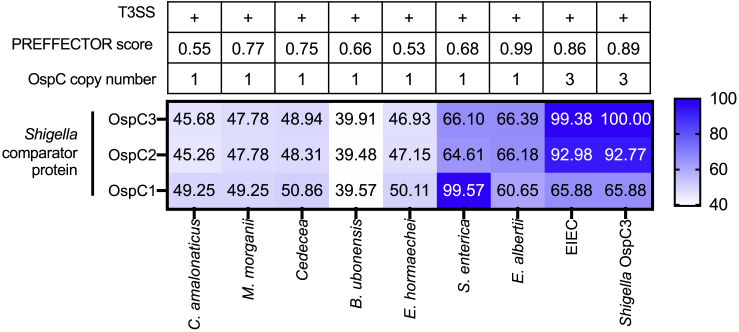
Pairwise comparison of *Shigella* OspC1, OspC2, and OspC3 with their identified homologs, related to Figure 5 The genomes harboring OspC homologs (Figure 5A) were subject to BLAST analysis to identify components of the type III secretion system (T3SS) translocon using the *Shigella* MxiD amino acid sequence as query. T3SS containing genomes are displayed with a plus symbol. Amino acid sequences of the OspC homologs were subject to analysis with PREFFECTOR, which searches for putative secretion sequences. The PREFFECTOR score is given in the table, with 1 being the maximum value, and 0 being the minimum. The number of BLAST hits in the OspC query was compared per genome as a proxy for OspC copy number, which is also indicated in the table. The OspC homologs identified by BLASTp in Figure 5A were subject to multiple sequence alignment using ClustalW against *Shigella* OspC1, 2, and 3 sequences. The output pairwise identity matrix was visualized in Prism, and percentage sequence similarity is visualized by a sliding color scale shown in the figure.

We sought to determine if OspC effectors were limited to *Shigella* spp*.* or whether they were conserved across multiple pathogens. A BLASTp query of the OspC3 amino acid sequence in the NCBI database revealed several homologs distributed across *Proteobacteria*. Phylogenetic analysis revealed that the most likely recent common ancestral sequences included plant-associated opportunistic human pathogens (Figure 5A). Furthermore, the *Shigella* OspC3 sequence was rooted in a clade consisting of homologs from the major human pathogens Enteroinvasive *Escherichia coli* (EIEC) (Pasqua et al., 2017) and *Salmonella enterica* (Figueira et al., 2013), in addition to the emerging pathogens *Enterobacter hormaechei* (Wenger et al., 1997) and *Escherichia albertii* (Ooka et al., 2012) (Figure 5A). BLAST analysis confirmed that all strains contained components of T3SSs, and further interrogation of the homologs amino acid sequences with PREFFECTOR demonstrated that all contained signatures of putative effector proteins, indicating their propensity for secretion (Figure S6).

Comparison of representative OspC homologs with the *Shigella* OspC sequence revealed high degrees of similarity in the N-terminal domain and, to a lesser extent, the C-terminal domain (Figures 5B and 5C). We thus hypothesized that these OspC homologs might bind CaM and block IFN signaling. To verify this, representative effectors from across the phylogeny were expressed in 293T cells and lysates were subjected to pulldowns with CaM beads. We found that almost all effectors were able to bind Apo-CaM (Figure 5D). A notable exception was the *S. enterica* protein that harbored a large insertion relative to other effectors, potentially altering CaM binding. Moreover, functional analysis revealed that proteins from bacteria which are not frequently associated with human or animal infections, did not block ISRE activation (Figure 5E, gray bars). However, homologs from pathogenic EIEC, *E. hormaechei*, and *E. albertii* blocked ISRE activation to levels consistent with *Shigella* OspC3 (Figure 5E, blue bars). This indicates that these OspC-like effectors may represent previously unrecognized virulence determinants. More importantly, these data show that CaM binding and subsequent inhibition of IFN by OspC effectors is a virulence strategy conserved across multiple bacterial pathogens.

### Type I and III IFNs restrict infection of *Shigella* in epithelial cells

**Figure S7:**
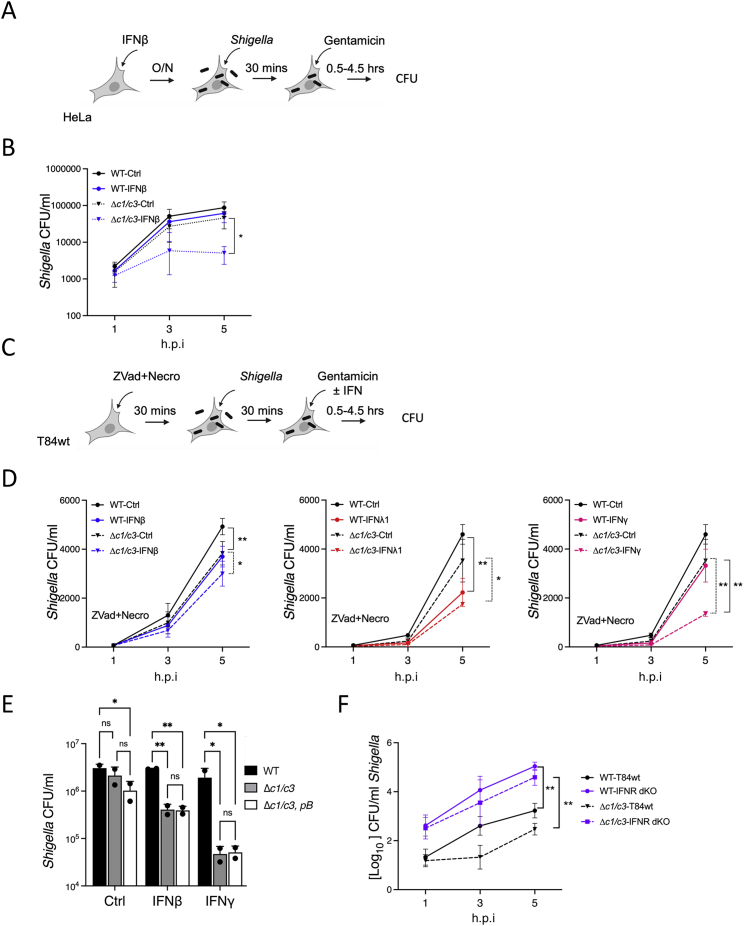
Restriction of *S. sonnei* infection by IFN is partially independent of cell death, related to Figure 6 (A) Experimental layout for (B). (B) HeLa cells were pretreated for 18 h with 10 ng/mL of IFNβ and then infected with *S. sonnei* (WT) or *ΔospC1/C3 Shigella* at a MOI of 100. 30 min later, cells were washed and treated with gentamicin. Infected cells were lysed and *Shigella* colony forming units (CFU) were enumerated at 1, 3, and 5 h postinfection (h.p.i). Data represent the mean ± SEM of five independent experiments. Statistical analysis was performed by one-way ANOVA. (C) Experimental layout for (D). (D) Wild-type T84 cells (T84wt) were treated with cell death inhibitors Z-VAD-FMK (ZVad) at 25 μM and Necrosulfonamide (Necro) at 20 μM 30 min before infection with *S. sonnei* (WT or *ΔospC1/C3*) at a MOI of 30. 30 min later, cells were washed and treated with gentamicin and 10 ng/mL of IFNβ (left), IFNλ1 (middle), or IFNγ (right). Inhibitors were kept through the duration of the experiment. *Shigella* colony forming units (CFU) were enumerated at 1, 3, and 5 h postinfection. Data represent the mean ± SEM of 2–4 independent experiments. Statistical analyses were performed by two-way ANOVA. (E) HeLa cells were pretreated for 18 h with 10 ng/mL of IFNβ or IFNγ. Cells were then infected with AfaI-expressing *S. sonnei* (WT), *ΔospC1/C3* or *ΔospC1/3, pB* (expressing an OspC3 variant that blocks IFN but does not induce cytotoxicity, MOI 50). Cells were lysed 5 h postinfection and CFUs were enumerated. Data represent the means ± SD of 2 independent experiments performed in triplicates. (F) Related to Figure 6E. 1 × 10^5^ T84wt or cells that lack type I and type III IFN receptors (IFNR dKO, purple lines) were infected with WT (plain lines) or Δ*ospC1/C3 Shigella* (dashed lines) at MOI 30. At the indicated time points, infected cells were lysed and *Shigella* CFUs were enumerated. Data represent the means ± SEM of at least 3 independent experiments. Statistical analyses were performed by two-way ANOVA. ^∗^ p < 0.05; ^∗∗^ p < 0.01.

Our data show that the highly conserved *Shigella* OspC family of effectors inhibits signaling downstream of type I and III IFNs. Evolution of such countermeasures suggests these IFNs impose a significant selection pressure against intracellular bacteria. We thus compared the growth of *Shigella* within epithelial cells in the presence and absence of IFNs via enumeration of intracellular bacterial colony forming units (CFUs) using gentamicin protection assays. Pretreatment of HeLa cells with IFNβ slightly inhibited the replication of intracellular WT *Shigella sonnei* (Figures S7A and S7B). Growth of Δ*ospC1/C3 Shigella* was moderately attenuated under control conditions and completely inhibited in the presence of IFNβ (Figure S7B).

**Figure 6:**
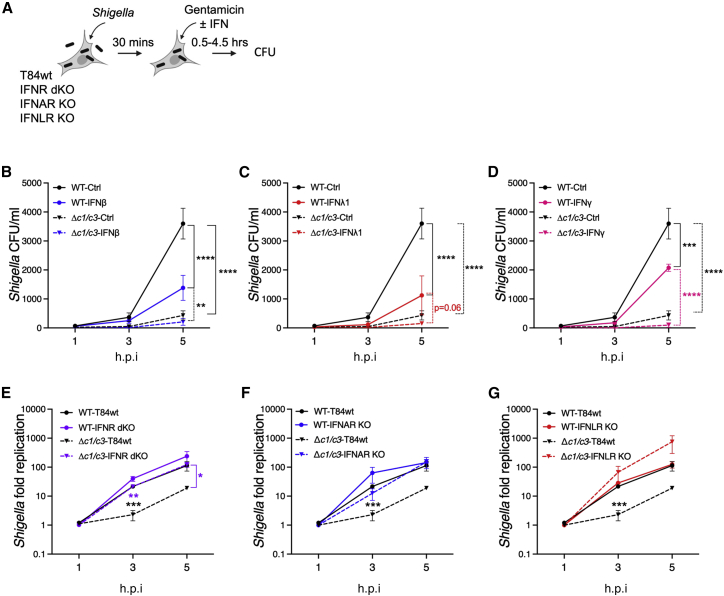
Type I and III IFNs restrict infection of *Shigella* in epithelial cells (A) Experimental layouts for (B)–(G) and S8F. (B–D) Wild-type T84 cells (T84wt) were infected with *S. sonnei* (WT or *ΔospC1/C3*) at MOI 30 and treated with IFNβ (B), IFNλ1 (C), or IFNγ (D). Cells were lysed, and *Shigella* colony forming units (CFU) were enumerated at 1, 3, and 5 h postinfection (h.p.i). (E–G) T84wt or cells that lack type I and type III IFN receptors (IFNR dKO, purple lines, E), type I IFN receptor (IFNAR KO, blue lines, F) or type III IFN receptor (IFNLR KO, red lines, G) were infected with WT (plain lines) or Δ*ospC1/C3 Shigella* (dashed lines) at MOI 30. *Shigella* CFUs were enumerated at the indicated time points and plotted as fold replication following normalization over the 1 h time point for each condition. Data show means ± SEM of at least 3 independent experiments. Statistical analyses were performed by two-way ANOVA. ^∗^ p < 0.05; ^∗∗^ p < 0.01; ^∗∗∗^ p < 0.001; ^∗∗∗∗^ p < 0.0001. See also Figure S7.

To determine the sensitivity of Δ*ospC1/C3 Shigella* to additional IFNs, we made use of T84 colonic epithelial cells (T84wt), which are responsive to all 3 IFN families (Pervolaraki et al., 2019). Cells were infected with WT or Δ*ospC1/C3 Shigella*. IFNβ, IFNλ1, or IFNγ was added 30 min postinvasion, allowing enough time for effector secretion and binding of OspCs to their host cell targets (Figures 6A–6D). Although each IFN treatment lowered WT *Shigella* replication, Δ*ospC1/C3 Shigella* were entirely unable to replicate in the presence of IFN (Figures 6B–6D).

OspC1 and OspC3 block the activities of Caspase 3/7 and Caspase 4/11, respectively. To investigate whether cell death induced by these Caspases plays a role in the observed IFN-mediated restriction of intracellular bacterial replication, we pretreated T84 cells with the broad-spectrum Caspase inhibitor Z-VAD-FMK and the necroptosis inhibitor, necrosulfonamide (Figures S7C and S7D). The growth of Δ*ospC1/C3 Shigella* was only partially restored in the presence of these inhibitors. This finding shows that the defect in growth of the mutant is partially due to its inability to block cell death, confirming previous reports (Ashida et al., 2020; Wandel et al., 2020). However, in the presence of these drugs, the intracellular growth of Δ*ospC1/C3* was still significantly restricted, and IFNs significantly further lowered bacterial numbers (Figure S7D). Similarly, the growth in HeLa cells of Δ*ospC1/C3*, *pB Shigella* that cannot block ISG expression (Figures 2G, 4F, and S3H) but can block host cell death (Figures S3E–S3G), was inhibited by IFNβ or IFNγ (Figure S7E) to levels undistinguishable from Δ*ospC1/C3* bacteria. These data demonstrate that IFN-mediated restriction of Δ*ospC1/C3 Shigella* replication is, in part, independent of host cell death.

Growth of Δ*ospC1/C3* in T84 cells was very limited in the absence of exogenous IFN, leading us to speculate that this growth inhibition is due to the activity of endogenously produced IFN. Therefore, we compared the replication of *Shigella* strains in WT T84 cells (T84wt) or cells lacking either the type I IFN receptor (IFNAR KO), the IFNλ receptor (IFNLR KO), or IFNAR and IFNLR double knockout (IFNR dKO) (Figures 6A, 6E–6G, and S7F). In T84wt cells, Δ*ospC1/C3 Shigella* replicated to significantly lower levels than WT bacteria (Figures 6E–6G, black stars). However, in IFNR dKO cells, Δ*ospC1/C3* bacteria were no longer replication defective compared with WT bacteria (p = 0.4) and replicated an order of magnitude more efficiently than in WT cells (purple stars). IFNAR (Figure 6F) or IFNLR KO cells (Figure 6G) displayed comparable phenotypes, with IFNLR deletion having the strongest effect.

### OspC effectors block IFN signaling to facilitate colonization of the murine intestine

**Figure 7:**
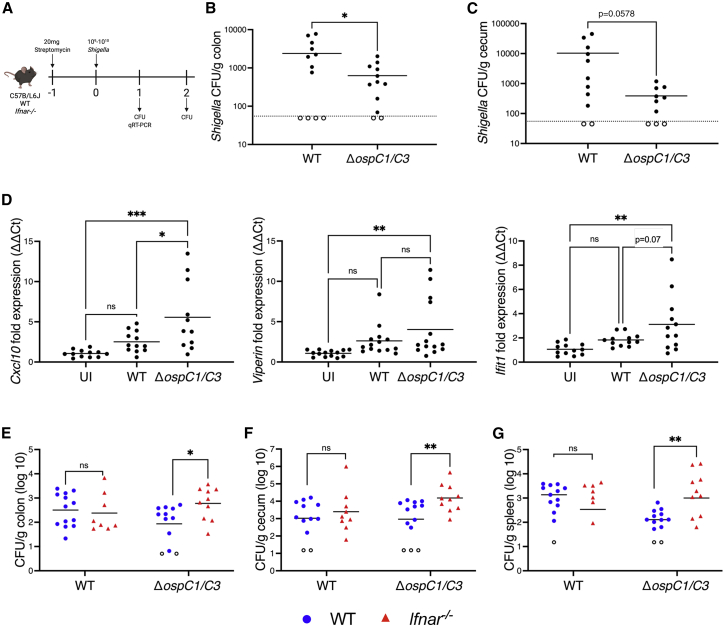
OspC effectors facilitate colonization of the murine intestine (A) WT or *Ifnar*^*−/−*^ C57B/L6J mice treated with 20 mg of streptomycin sulfate were orally challenged the next day with streptomycin-resistant WT or Δ*ospC1/C3 Shigella*. Endpoint harvest was performed at day 1 or 2 postinfection. (B and C) At day 1 postinfection, CFU were determined from the colon (B) and cecum (C) of WT mice. (D) *Cxcl10*, *Viperin*, and *Ifit1* mRNA expression in the colon of uninfected (UI) or *Shigella*-infected WT mice were determined by qRT-PCR and calculated using the ΔΔCt method. Statistical significance was determined with one-way ANOVA. (E–G) Similarly to (B) and (C) except CFU were determined from colon, cecum, and spleen of WT or *Ifnar*^*−/−*^ deficient mice 2 days postinfection. Data were not normally distributed, so were Log_10_ transformed prior to analysis. (B, C, and E–G) Statistical analysis was performed using unpaired Student’s t test. Symbols represent individual animals. Samples below the limit of detection are indicated by unfiled symbols. ns, nonsignificant; ^∗^ p < 0.05; ^∗∗^ p < 0.01; ^∗∗∗^ p < 0.001. See also Figure S8.

**Figure S8:**
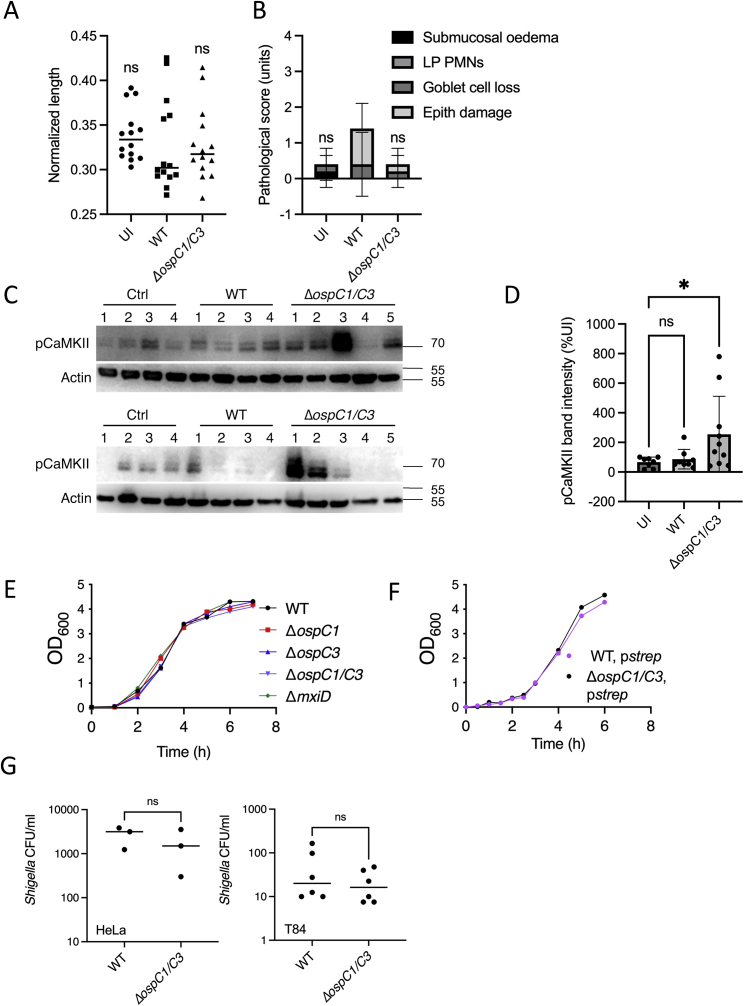
WT *Shigella* infection does not lead to inflammation but blocks CaMKII phosphorylation *in vivo*, related to Figure 7 and STAR Methods (A) Quantification of colon lengths in mice infected with WT or *ΔospC1/C3 S. sonnei*. Values were normalized to mouse weight prior to infection; colon length (cm)/mouse weight (g). UI, uninfected (circle symbols); WT, wild-type *S. sonnei* (square symbols); *ΔospC1/C3* (triangle symbols). Data are representative of 3 experiments. Each symbol represents a mouse. (B) Blinded quantification of histology score from one representative experiment. Submucosal edema; LP PMNs, lamina propria polymorphonuclears; Goblet cell loss; Epith damage, epithelial damage, were scored from 0 to 4. The final score is the sum of individual scores from each category. Statistical analysis was performed by one-way ANOVA, comparing each condition to WT-infected condition. ns, nonsignificant. Data show mean ± SD of 1 experiment representative of 2. (C) Colon samples from mice infected with WT or *ΔospC1/C3 S. sonnei* were subjected to SDS-PAGE and western immunoblotting against phosphorylated CaMKII. Data shown are from 2 different experiments. Numbers represent individual mice for each condition. (D) Quantification of experiment shown in (C). Each symbol represents a mouse. Statistics were performed by one-way ANOVA, comparing each condition to uninfected (UI). Data represent the means ± SD of 8–10 mice per condition. (E and F) Native (E) and streptomycin-resistant (F) strains used in this study were grown in tryptic soy broth (TSB). OD_600_ was measured at the time points indicated. (G) HeLa (left panel) or T84 (right panel) epithelial cells were infected with wild type (WT) or *ΔospC1/C3 Shigella* at MOI 100 or 30 respectively. 30 min later, cells were washed and treated with gentamicin for an additional 30 min. *Shigella* colony forming units (CFU) were enumerated at 1 h postinfection. Statistical analysis was performed by Student’s t test. ns, nonsignificant; ^∗^ p < 0.05.

Although *S. sonnei* causes limited disease pathology when administered orally to mice, it is able to colonize the intestines of mice pretreated with streptomycin (Anderson et al., 2017). Thus, we orally inoculated WT C57BL/6J mice with phosphate buffered saline (PBS), streptomycin-resistant WT, or Δ*ospC1/C3 Shigella* for 24 h (Figure 7A). Consistent with previous observations (Anderson et al., 2017; Singer and Sansonetti, 2004), colon length and inflammation were similar to uninfected animals (Figures S8A and S8B). Pathological scores revealed very minor epithelial damage and goblet cell loss in infected mice compared with uninfected (Figure S8B). After gentamicin treatment to clear extracellular organisms, bacterial CFUs were plated to quantify intracellular bacterial burdens. Bacterial titers in the colon of WT-infected mice were significantly higher than those of Δ*ospC1/C3*-infected colons (Figure 7B). CFUs in the cecum showed the same trend (Figure 7C) with almost a log difference between the two strains, although this difference was not statistically significant (p = 0.0578).

We next sought to determine whether CaMKII phosphorylation and ISG expression was increased in mice infected with Δ*ospC1/C3* bacteria. At 24 h postinfection, comparable phosphorylation of CaMKII was found in the colons of uninfected and WT *Shigella*-infected mice, whereas those of mice infected with the Δ*ospC1/C3* strain exhibited significant higher levels of pCaMKII (Figures S8C and S8D), suggesting that the lack of OspC1 and OspC3 increases CaMKII phosphorylation in mouse colonic cells. qRT-PCR analysis revealed that WT *Shigella* infection induced a small but nonsignificant expression of multiple ISGs (*Cxcl10*, *Viperin*, and *Ifit1*) in the colon compared with uninfected organs (Figure 7D). Conversely, infection with the Δ*ospC1/C3* strain induced a significant increase in expression of these 3 ISGs in the colon compared with uninfected animals (Figure 7D). Moreover, *Cxcl10* expression was significantly increased in mice infected with Δ*ospC1/C3 Shigella*, compared with WT *Shigella*, despite the lower bacterial titers observed in the Δ*ospC1/C3* infection. Together these data demonstrate that OspC1 and OspC3 predispose *Shigella* to efficient infection of the tissue, an observation that correlates with increased expression of antibacterial ISGs in Δ*ospC1/C3*-infected animals.

The role of IFNs in bacterial infection is complex. Notably, type I IFNs are either protective or detrimental to the host depending on the bacterial species and disease model (Boxx and Cheng, 2016; Peignier and Parker, 2021). Consequently, we deciphered the role of type I IFNs in anti-*Shigella* immunity by infecting WT and *Ifnar*^−/−^ mice with WT and Δ*ospC1/C3 S. sonnei*. The colonization defect of Δ*ospC1/C3 Shigella* in the colon and cecum of WT mice was rescued in the *Ifnar*^−/−^ background (Figures 7E and 7F). Similarly, although Δ*ospC1/C3* displayed reduced dissemination to the spleen of WT mice, this deficiency was rescued in *Ifnar*^−/−^ mice (Figure 7G). Together, these data highlight the crucial role that IFNs play in intestinal defense against *Shigella*. In response, OspC effectors are indispensable for *Shigella* to subvert host responses and colonize the murine gut.

## Discussion

The functions of type I and III IFNs in antibacterial immunity remain poorly understood. Depending on the pathogen and route of infection, type I IFNs have been shown to be protective or detrimental to hosts infected with bacteria (Alphonse et al., 2021; Boxx and Cheng, 2016; Peignier and Parker, 2021). We previously showed that type III IFNs protect epithelial barriers against invasive bacteria (Odendall et al., 2017), but the mechanisms by which type III IFNs afford protection remain unknown. Here, we find that both type I and III IFNs restrict *S. sonnei* infection of epithelial cells. Most importantly, our data reveal a role for type I IFN in protecting against *Shigella* colonization in a mouse model of infection.

The importance of type I and III IFNs in immunity against bacteria is underlined by our discovery that *Shigella* blocks IFN signaling and ISG expression. Our multilayered approach encompassing global and targeted techniques revealed that *Shigella* utilizes the OspC family of effectors as potent inhibitors of the host IFN response. Although most viruses possess at least one virulence factor that blocks signaling downstream of IFN receptors, our study reports identification of bacterial effectors that inhibit JAK/STAT and IFN signaling and ISG expression. Our findings establish both the importance of IFN inhibition in bacterial pathogenesis and re-enforce the role of this family of cytokines in antibacterial immunity.

Regulation of IFN signaling is multifaceted, involving the coordinated activity of multiple host proteins (Stanifer et al., 2019). In this study, we demonstrate a previously undescribed mechanism by which OspC1 and OspC3 block IFN signaling through their interaction with the ubiquitous Ca^2+^ sensor CaM, preventing it from binding and activating its key target CaMKII. Intriguingly, although we detected a very clear interaction between OspC2 and Apo-CaM, OspC2 did not block CaMKII phosphorylation. Future structural determination of Apo-CaM/OspC complexes will decipher the molecular determinants of CaM inhibition by OspC1 and OspC3 and shed light into OspC2’s divergent activity. Furthermore, inhibition of CaMKII by OspC1 and OspC3 blocked STAT1 phosphorylation, a phenotype that was phenocopied by pharmacological inhibition of CaM/CaMKII. Our data confirm a previously described (Nair et al., 2002; Wang et al., 2008) but underappreciated role of the Ca^2+^/CaM/CaMKII axis in modulating IFN signaling and ISG expression, highlighting the importance of this pathway in controlling bacterial infections.

OspC1 and OspC3 bind CaM and inhibit IFN via their N-terminal domain, independently of the residues and biochemical activity that mediate inhibition of cell death. These data not only reinforce the link between CaM targeting and IFN signaling inhibition but also demonstrate that OspC3 (and presumably OspC1) has at least two genetically and spatially separable functions. Other bacterial effectors have been attributed multiple functions, but our study highlights a rare example of multifunctional effectors with discrete molecular domains that mediate binding to distinct targets, as well as targeting of distinct host cell processes. These findings highlight the pleiotropic and pivotal role of OspC1 and OspC3 in *Shigella* pathogenesis.

Our data also revealed the presence of OspC-like effectors in diverse species of bacteria. Fascinatingly, although CaM binding is an ancestral trait of most OspC homologs tested, inhibition of IFN responses was restricted to the pathogens EIEC, *E. albertii*, and *E. hormaechei*. This not only demonstrates that inhibition of Ca^2+^, and by extension IFN signaling, is a widespread virulence strategy but that the ability to inhibit IFN may be a trait evolved in response to selection for immune evasion. Evidence is emerging that different OspC family members may leverage CaM binding for distinct functions. Indeed, *Chromobacterium violaceum* CopC, an OspC homolog, was recently shown to hijack CaM, using it as a cofactor to enable its enzymatic activity (Peng et al., 2022; Liu et al., 2022), similarly to *Bordetella* CyaA (Guo et al., 2005) or *Legionella* SidJ (Bhogaraju et al., 2019; Gan et al., 2019; Springer et al., 2017; Sulpizio et al., 2019). Although CopC possesses the same ADP-riboxanase activity as *Shigella* OspCs, it is unable to inactivate Caspases in the absence of CaM (Peng et al., 2022). OspC3 modifies Caspase 4 when both proteins are expressed in bacteria, in the absence of any eukaryotic protein (Li et al., 2021). Therefore, whether CaM is a *Shigella* OspC cofactor remains unclear. Our findings that OspC1 and OspC3 block CaMKII phosphorylation *in vitro* and *in vivo* argue that these bacterial effectors do inhibit CaM to target innate responses. These findings place CaM as a key molecular target for virulence factors conserved across multiple bacterial taxa, both by hijacking it to enable their function and by blocking its downstream targets, including those regulating innate immunity. The work presented in this study mandates further research into the roles of Ca^2+^ and CaM at the host-pathogen interface.

### Limitations of the study

This study examined the molecular basis for inhibition of IFN signaling, by a single family of homologous effector proteins. Future studies should investigate the combined activities of the multiple IFN inhibitory effector proteins identified in our IFN inhibitor screen under infection conditions. Further, although we demonstrate that deletion of OspC1 and OspC3 from *Shigella* renders them deficient in colonization of the murine intestine in WT mice, neither these mice nor the *Ifnar*^−/−^ mice displayed pathological signs of *Shigella* infection. Future studies utilizing NLRC4-deficient mice—a model of murine shigellosis (Mitchell et al., 2020)—will decipher the role of OspC effectors and by extension IFN signaling in *Shigella* pathogenesis. Finally, although AlphaFold predictions formed a conceptual framework for this study, future work should experimentally resolve the structure of the CaM-OspC complex.

## STAR★Methods

### Key resources table

**Table:** 

REAGENT or RESOURCE	SOURCE	IDENTIFIER
**Antibodies**		

anti-pSTAT1 (Y701)	BD Biosciences	Cat#612133; RRID:AB_399504
anti-STAT1	Cell Signaling	Cat#9172S; RRID:AB_2198300
anti-CaMKII	Cell Signaling	Cat#3362S; RRID:AB_2067938
anti-pCaMKII⍺	Cell Signaling	Cat#12716S; RRID:AB_2713889
anti-IFIT1	Cell Signaling	Cat#14769S; RRID:AB_2783869
anti-Viperin	MerkMillipore	Cat#MABF106; RRID:AB_11203644
anti-IFITM3	Proteintech	Cat#11714-1-AP; RRID:AB_2295684
anti-GFP	Chronotek	Cat#3h9-100; RRID:AB_10773374
anti-β-actin-HRP	Sigma	Cat#A5441; RRID:AB_476744
APC anti-human CD317 (Tetherin)	BioLegend	Cat#348410; RRID:AB_2067121
HRP conjugated anti-mouse IgG	Jackson	Cat#115-035-146; RRID:AB_2307392
Dylight 800 conjugated anti-mouse IgG	Cell Signaling	Cat#5257S; RRID:AB_1069354
HRP conjugated anti-rabbit IgG	Jackson	Cat#111-035-003; RRID:AB_2313567
HRP conjugated anti-rat IgG	Jackson	Cat#712-035-150; RRID:AB_2340638

**Bacterial and virus strains**		

Shigella sonnei 53G	Caboni et al. (2015)	N/A
Shigella sonnei 53GΔospC1	This study	N/A
Shigella sonnei 53GΔospC3	This study	N/A
Shigella sonnei 53GΔospC1/3	This study	N/A
Shigella sonnei 53GΔmxiD	Watson et al. (2018)	N/A
Shigella sonnei 53G - StrpR	This study	N/A
Shigella sonnei 53GΔospC1/3 – StrpR	This study	N/A
Shigella sonnei 53G - AfaI	This study	N/A
Shigella sonnei 53GΔospC1 - AfaI	This study	N/A
Shigella sonnei 53GΔospC3 – AfaI	This study	N/A
Shigella sonnei 53GΔospC1/3 – AfaI	This study	N/A
Shigella sonnei 53GΔmxiD - AfaI	This study	N/A
Shigella sonnei 53GΔospC1/3,pB - AfaI	This study	N/A

**Chemicals, peptides, and recombinant proteins**		

IFNα	Pbl Assay Science	Cat#11100-1
IFNβ	Peprotech	Cat#300-02BC
IFNλ1	Peprotech	Cat#300-02L
IFNγ	Peprotech	Cat#300-02
mIFNβ	R&D systems	Cat#12405-1
Z-VAD-FNK	Apexbio	Cat#A1902
Necrosulfonamide	Tocris	Cat#5025
W7	Calbiochem	CAS 61714-27-0
KN93	MedChemExpress	HY-15465
TyrA9	Calbiochem	CAS 10537-47-0

**Critical commercial assays**		

CyQUANT LDH Cytotoxicity Assay	Thermo Scientific	Cat#C20301
CellTiterGlo luminescence detection assay	Promega	Cat#G7570
TaqMan RNA-to-Ct 1-step kit	Applied Biosystems	Cat#4392938

**Deposited data**		

RNAseq raw and analyzed data	This paper	GSE200447

**Experimental models: Cell lines**		

HEK293T-ISRE luciferase cells	N. Hacohen (Massachusetts General Hospital)	N/A
HEK293T	ATCC	ATCC CRL-1573
HeLa	ATCC	ATCC CCL-2
Swiss 3T3 cells	David Holden (Odendall et al., 2012)	N/A
T84 wt	Steeve Boulant and Megan Stanifer	N/A
T84 IFNAR KO	Steeve Boulant and Megan Stanifer	N/A
T84 IFNLR KO	Steeve Boulant and Megan Stanifer	N/A
T84 IFNR dKO	Steeve Boulant and Megan Stanifer	N/A
HeLa Tet-Off control cells	TakaraBio	Cat# 631156
CaM knock-out HeLa cells (Clone 5B5)	Munk et al., 2020	N/A

**Experimental models: Organisms/strains**		

C57BL/6J mice	Charles River or Major et al. (2020)	N/A
*Ifnar1*^*−/−*^ C57BL/6J mice	Major et al. (2020)	N/A

**Oligonucleotides**		

OspC1_LRR_F: 5’attaaaactgttttcatataaggtt cattttatgaatatagtgtaggctggagctgcttc3’	Integrated DNA Technologies	N/A
OspC1_LRR_R: 5’ctgccttttgctaaacgatattca attttgattaaatatacatatgaatatcctccttag3’	Integrated DNA Technologies	N/A
OspC3_LRR_F: 5’cagttagataatgttatctaaata accacagataaaaacgcacataattgcatatgaatatc ctccttag3’	Integrated DNA Technologies	N/A
OspC3_LRR_R: 5’gggacagaatcactcatgatgac ttcgataatcgacgacattattatttggtgtaggctggagct gcttc3’	Integrated DNA Technologies	N/A
*RSAD2 (VIPERIN)*	ThermoFisher	Cat#4331182 Hs00369813_m1 *RSAD2* FAM
*IFIT1*	ThermoFisher	Cat#4331182 Hs01911452-s1 IFIT1 FAM
*GAPDH*	ThermoFisher	Cat#4331182 Hs00266705-g1 *GADPH* FAM
*Rsad2 (Viperin)*	ThermoFisher	Cat#4331182 Mm00491265-m1 *Rsad2* FAM
*Ifit1*	ThermoFisher	Cat#4331182 Mm00515153_m1 *Ifit1* FAM
*Cxcl10*	ThermoFisher	Cat#4331182 Mm00445235_m1 *Cxcl10* FAM
*Gapdh*	ThermoFisher	Cat#4331182 Mm99999915_g1 *Gapdh* FAM

**Recombinant DNA**		

pKD4	Datsenko and Wanner (2000)	N/A
pKD46	Datsenko and Wanner (2000)	N/A
pCP20	Datsenko and Wanner (2000)	N/A
pBR322-AfaI	Labigne-Roussel et al. (1984)	N/A
pRL1383a	Wolk et al. (2007)	N/A
pB-puc57	This study	N/A
pEGFP-C1	Clontech	Cat#6084-1
pDsRED-monomer-C1	Clontech	Cat#632466
pEGFP-C1-ccdB	Sandstrom et al. (2019)	N/A
pDsRED-monomer-C1-ccdB	This study	N/A
pEGFP-C1 / pDsRed-C1 – OspC1	This study	N/A
pEGFP-C1 / pDsRed-C1 – OspC2	This study	N/A
pEGFP-C1/ pDsRed-C1 – OspC3	This study	N/A
pEGFP-C1 / pDsRed-C1 – OspD1	This study	N/A
pEGFP-C1 – IcsB	This study	N/A
pDsRed-C1 – IpaH1.4	This study	N/A
pEGFP-C1 – IpaH1.4	Sandstrom et al. (2019)	N/A
pEGFP-C1 – IpaH4.5	Sandstrom et al. (2019)	N/A
pEGFP-C1 – IpaH7.8	Sandstrom et al. (2019)	N/A
pEGFP-C1/ pDsRed-C1 – IpgB2	This study	N/A
pEGFP-C1 – IpaB	This study	N/A
pEGFP-C1 – IpaC	This study	N/A
pEGFP-C1 – IpaD	This study	N/A
pEGFP-C1 – IpaJ	This study	N/A
pEGFP-C1 – IpgB	This study	N/A
pEGFP-C1 – OspB	This study	N/A
pEGFP-C1 – OspD1	This study	N/A
pEGFP-C1 – OspD2	This study	N/A
pEGFP-C1–OspD3	This study	N/A
pEGFP-C1 / pDsRed-C1 – OspE1	This study	N/A
pEGFP-C1 OspF	This study	N/A
pEGFP-C1 / pDsRed-C1 – VirA	This study	N/A
pEGFP-C1/ pDsRed-C1 – NSP2	This study	N/A
pCAG-mEGFP-CaMKIIa (T286D/T305A/T306A)	Chang et al. (2019)	N/A
pGL4.45 ISRE-Luc	Promega	Cat#9PIE414
pGAS/ISRE-Luc	Biocat.com	Cat#LR-2016-SO
pEGFP-C1 – OspC1-N_t_	This study	N/A
pEGFP-C1 – OspC1-C_t_ C	This study	N/A
pEGFP-C1 – OspC2-N_t_	This study	N/A
pEGFP-C1 – OspC2-C_t_ C	This study	N/A
pEGFP-C1 – OspC3-N_t_	This study	N/A
pEGFP-C1 – OspC3-C_t_	This study	N/A
pEGFP-C1 – OspC2/3 A	This study	N/A
pEGFP-C1 – OspC2/3 B	This study	N/A
pEGFP-C1 – OspC2/3 C	This study	N/A
pEGFP-C1 – OspC2/3 D	This study	N/A
pEGFP-C1 – OspC3/2 E	This study	N/A
pEGFP-C1 – OspC3/2 F	This study	N/A
pEGFP-C1 – OspC3/2 G	This study	N/A
pEGFP-C1 – OspC3/2 H	This study	N/A
pEGFP-C1 – OspC-*Citrobacter*	This study	N/A
pEGFP-C1 – OspC-*Morganella*	This study	N/A
pEGFP-C1 – OspC-*Cedecea*	This study	N/A
pEGFP-C1 – OspC-*Burkholderia*	This study	N/A
pEGFP-C1 – OspC-*Enterobacter*	This study	N/A
pEGFP-C1 – OspC-*Salmonella*	This study	N/A
pEGFP-C1 – OspC-*Escherichia albertii*	This study	N/A
pEGFP-C1 – OspC-EIEC	This study	N/A
pEGFP-C1-OspC1-EH/AA	This study	N/A
pEGFP-C1-OspC3- EH/AA	This study	N/A
pRL-CMV-Renilla	Promega	Cat#E2231

**Software and algorithms**		

Fiji	Fiji v 2.1.0	RRID: SCR_002285
FlowJo	FlowJo v 10	RRID: SCR_008520
ImageJ	Schindelin et al. (2012)	https://imagej.nih.gov/ij/
Alphafold	Jumper et al. (2021)	N/A
Nfcore/rnaseq pipeline	V 3.5 (Ewels et al., 2020)	N/A (Ewels et al., 2020)
Nextflow domain specific language	V 19.10.0 (Di Tommaso et al., 2017)	N/A (DI Tommaso et al., 2017)
Singularity	V 2.6.0 (Kurtzer et al., 2017)	N/A(Kurtzer et al., 2017)
RSEM-STAR	Dobin et al. (2013), Li and Dewey (2011)	(Dobin et al., 2013; Li and Dewey, 2011) N/A
DESeq2	v1.28.0 (Love et al., 2014)	RRID: SCR_015687
R package Cluster Profiler	v3.18.1 (Yu et al., 2012)	RRID: SCR_016884
Molecular Signatures database	MSigDB, v7.2	RRID: SCR_016863

### Resource availability

#### Lead contact

Further information and requests for resources and reagents should be directed to and will be fulfilled by the lead contact, Charlotte Odendall (charlotte.odendall@kcl.ac.uk).

#### Materials availability

All unique/stable reagents generated in this study are available from the lead contact without restriction.

### Experimental model and subject details

#### Cell culture and treatments

HEK293T-ISRE luciferase cells were obtained from N. Hacohen (Massachusetts General Hospital). HEK293T and HeLa cells were obtained from ATCC and were routinely cultured in Dulbecco’s Modified Eagle Medium (DMEM; Thermo Fisher) supplemented with 10% Fetal Bovine Serum (FBS) 1% of penicillin/streptomycin (Thermo Fisher) at 37°C supplemented with 5% CO_2_. Swiss 3T3 cells were a kind gift from David Holden (Odendall et al., 2012). T84 wt, IFNAR KO, IFNLR KO and IFNR dKO human colon carcinoma cells (Pervolaraki et al., 2017) were kindly provided by Steeve Boulant and Megan Stanifer and were cultured in DMEM+F12 medium supplemented with 10% FBS, 1% of penicillin/streptomycin at 37°C supplemented with 5% CO_2_. Conditional CaM knock-out Hela cells (Clone 5B5) and their Tet-OFF parental cells were routinely cultured in DMEM supplemented with quality controlled tetracycline free FBS (Gibco) as previously described (Munk et al., 2020). Prior to use, cells were cultured in 1 μg/mL Doxycycline (Sigma) for 72 hrs to induce CaM depletion.

Unless stated otherwise, when indicated, cells were treated with 10 ng/mL of human IFNβ, λ1 or γ (Peprotech), 1.6-1000 U/m of human IFNα (Pbl Assay Science) or 1000 U/mL of mouse IFNβ. The following drugs were used and unless otherwise stated were applied to cells 30 mins prior to infection until the experimental endpoint; Z-Val-Ala-Asp-fluoromethylketon (Z-VAD-FMK) (25 μM, Apexbio), Necrosulfonamide (20 μM, Tocris), W7 (CaM inhibitor, 2.5-20 μM, Calbiochem), KN93 (CaMKII inhibitor, 2.5-20 μM, MedChemExpress), TyrA9 (Pyk2 inhibitor, 0.625-5 μM, Calbiochem).

#### Bacterial strains and culture conditions

This study utilized *Shigella sonnei* strain 53G and its isogenic deletion mutants for *ospC1, ospC3*, and *mxiD*. All bacterial strains used and generated in this study are outlined in the key resources table. In frame deletion mutants were generated using the lambda red recombination system as previously described (Datsenko and Wanner, 2000), using primers outlined in the key resources table. Plasmids were transformed into competent *Shigella* strains by electroporation using a Biorad Micropulser. All *Shigella* strains were grown in trypticase soy broth (TSB, Sigma) at 37°C with 200 rpm shaking, unless otherwise stated. Antibiotics used for selection were as follows; ampicillin (100 ug/mL), kanamycin (50 ug/mL), chloramphenicol (25 ug/mL) and streptomycin (100 ug/mL). MOIs are indicated on each figure legend. For experiments using the Δ*ospC1/C3 Shigella* complimented with the pB OspC3/2 chimera (Δ*ospC1/C3, pB*), strains were grown in Lysogeny Broth (LB) lacking glucose to alleviate repression of the *lac* promoter. Growth in culture medium and invasion of Hela and T84 cells was determined for all recombinant *Shigella* strains prior to use in this study (Figures S8E–S8G).

#### Oral murine infection model

Oral murine infections were performed in male and female 6-16-week-old C57BL/6J and *Ifnar1*^*−/−*^mice. All animals used in this study were handled in accordance with the Home Office, UK project license P292BBCE, under the Animals (Scientific Procedures) Act 1986. Procedures and experiments were approved by the King’s College London animal ethic committee. C57/BL/6J mice were either obtained from Charles River or bred at the King’s College facility under pathogen-free condition. *Ifnar1*^*−/−*^ mice were bred at the King’s College facility under pathogen-free condition. Groups of 4-6 mice were randomly allocated and maintained in a 7am-7pm light cycle, according to standard husbandry practices at the Kings College London Biological Safety Unit. During experiments, female C57BL/6J and *Ifnar1*^*−/−*^ mice were co-housed in the same cage. Mice starved for 3 hrs were orally gavaged with 200 μL of 100 mg/mL streptomycin sulphate (20 mg/mouse) and placed in a cage with fresh bedding. 24 hrs later, mice were again starved for 3 hrs before being orally gavaged with 10^9^-10^10^ CFU of log-phase, streptomycin resistant WT or *ΔospC1/C3 S. sonnei* 53G, resuspended in 400 μL PBS. Mouse weights and sign of diseases were recorded at regular intervals. Mice were culled, and aseptically removed and processed post-mortem. Infection inputs were verified by serially diluting a fraction of the initial inoculum and plating on TSB plates containing 0.01% Congo red (CR) and 100μg/mL streptomycin.

### Method details

#### Plasmids and eukaryotic transfection

Each effector mammalian expression plasmid was constructed using the Gateway™ recombination system (Invitrogen). Entry plasmids for the full-length effectors were generated as previously described (Schmitz et al., 2009). Entry plasmids for the OspC2/OspC3 chimeras were generated via sewing overlap PCR using oligomers described in the key resources table. Genes encoding the chimeras were introduced into pDNR221 and sequence verified. The full length and chimeric effector genes were transferred into gateway compatible variants of pEGFP-C1 and pDsRed-monomer-C1 (Clontech). These Gateway compatible variants were generated by introducing a Gateway cassette into the *SmaI* restriction site in the polylinker of each parent vector. Additional transfection vectors expressing OspC homologs from *Citrobacter amalonaticus*, *Burkholderia ubonensis*, *Cedecea spp.*, *Morganella morganii*, *Escherichia albertii*, *Enterobacter hormaechei*, Enteroinvasive *E. coli* (EIEC), and *Salmonella enterica*, in addition to catalytically inactive *Shigella* OspC EH/AA in the pEGFP-C1 background were synthesized by Biobasic (Canada).

All transfection plasmids used in this study are outlined in the key resources table. Cells were transfected using jetPRIME (Polyplus) or PEI (PolySciences) according to the manufacturer’s instructions. Successful transfection was monitored by way of GFP fluorescence using a Floid Cell Imaging Station (Life Technologies).

#### ISRE reporter luciferase assay

HEK293T cells were seeded at a density of 2x10^5^ cells/mL and were transfected as above with the pGL4.45 ISRE-Luc reporter plasmids and incubated overnight (O/N). Where indicated, a Renilla internal control was used to account for changes in cell viability. Alternatively, 293T-ISRE luciferase cells were used. The following day, cells were treated O/N with IFNs for 18 hrs at the indicated concentrations before lysis in Lysis buffer (3.3g Gly-Gly, 1.8g MgSO_4_ anhydrous, 1.52g EGTA tetrasodium, 2.5mL Triton-100X, pH 7.8) + BrightGlo (Promega) containing the luciferin substrate for the reaction. If using Renilla, a dual Firefly/Renilla assay kit was used (Promega), following the manufacturers recommendation. Samples were transferred to white flat bottom plates (Biovision), and luminescence was read using a PerkinElmer 2030 VICTOR X Light plate reader.

#### Gentamicin protection assay

Cells were seeded at 2x10^5^ cells/mL in antibiotic-free complete medium. Where indicated, cells were treated with IFNs or drugs once they had adhered to the well. The following day, cells were starved in antibiotic free medium with 1% FBS for 2 hrs, before challenge with logarithmic phase (OD_600_= 0.8-1.0) bacteria at the indicated multiplicity of infection for 30-45 mins at 37°C. Cells were then washed three times in PBS and placed in medium containing 10% FBS supplemented with 100 μg/mL of gentamicin and incubated for another 30 mins (1 h post infection) to 4.5 hrs (5 hrs post infection) at 37°C. At the respective time points, cells were washed and lysed in PBS (Thermo Fisher) containing 1% Triton100X (ChemCruz). Lysates were serially diluted, and plated onto agar plates and the number of internalized bacteria was determined by counting the colony-forming units after O/N incubation at 37°C.

#### Western blotting

Protein extraction was performed using modified Radioimmunoprecipitation assay (RIPA) buffer (50 nM Tris-HCl, 1mM EDTA, 0.5mM EGTA, 1% NP-40, 0.25% sodium deoxycholate, 0.1% SDS, 250 mM NaCl) supplemented with phosphatase-phostop (Roche) and Halt Protease inhibitor (Nalgene). Electrophoresis was carried out on TGX gels (Biorad) using 1X Tris-Glycine running buffer. Proteins were transferred to a polyvinylidene difluoride membrane (Millipore) using BioRad Transfer Blot turbo system. Membranes were subsequently blocked with 5% milk in TBST for 1 h at room temperature. Primary antibody incubations were carried out either O/N at 4°C or for 2 hr at RT. Secondary antibody incubations were carried out for 1 hr at RT after 3x 5 mins washes in TBST. Blots were developed with Pierce™ ECL Plus western Blotting substrate, and detected on AMERSHAM ImageQuant 800 (GE Healthcare Life Sciences) or Odyssey (LI-COR). All primary and secondary antibodies used in this study are shown in the key resources table. Western blotting data were quantified using the ImageJ densitometry function comparing the protein of interest to loading control bands as previously described (Schindelin et al., 2012).

#### RNA extraction and qRT-PCR

Cells were lysed in RLT Lysis buffer (Qiagen 1015762) supplemented with β-mercaptoethanol. RNA was then isolated using RNA Mini Kit columns (Qiagen) following the manufacturer’s recommendations. qRT-PCR was carried out using TaqMan RNA-to-Ct 1-step kit (Applied Biosystems cat. 4392938) on the QuantStudio 5 (Applied Biosystems). Data were normalized against the expression of a control gene (GAPDH) followed by the untreated sample using the ΔΔCt method. When no counts were detected in a sample, an arbitrary number of 45 cycles was selected. All probes used in this study are shown in key resources table.

#### Flow cytometry assay

Cells were washed and detached from the tissue culture plate by incubating in TrypLE Dissociation Reagent (Gibco) for 5 mins at 37°C, transferred to 1.5mL conical tubes, pelleted by centrifugation at 500 x g for 5 mins at 4°C, stained in 100μl of APC-anti-CD317 antibody for 20 mins at RT and 1mL of Live/Dead dye (Invitrogen L34976A) for another 30 mins at RT, washed and resuspended in FACS buffer (1%BSA+0.1%sodium azide in DPBS). Samples were run on the FACS Aria (BD) flow cytometer equipped with the 488nm and 635nm lasers. Data were analyzed with FlowJo Software.

#### RNA-sequencing

RNA from HEK293T cells was extracted as above. Sequencing was performed on the NovaSeq platform and typically generated ∼25 million bp reads per sample. The nf-core/rnaseq pipeline (version 3.5; Ewels et al., 2020) written in the Nextflow domain specific language (version 19.10.0; Di Tommaso et al., 2017) was used to perform the primary analysis of the samples in conjunction with Singularity (version 2.6.0; Kurtzer et al., 2017). All data was processed relative to the human GRCh38 genome downloaded from Ensembl (release 95). Gene counts per gene per sample were obtained using the RSEM-STAR (Dobin et al., 2013; Li and Dewey, 2011) option of the pipeline and they were imported on DESeq (v1.28.0; Love et al., 2014) within R environment v4.0.3 for differential expression analysis. Gene Set Enrichment analysis (GSEA) was carried out using R package Cluster Profiler (v3.18.1; Yu et al., 2012) and gene lists ranked using the Wald statistic. Pre-ranked analyses were carried out using Hallmark pathway gene sets from the Molecular Signatures database (MSigDB, v7.2). Gene signatures were considered significant if FDR q-value ≤ 0.05.

#### Pull down assays

5x10^6^ 293T cells expressing GFP tagged proteins of interest were washed in cold TBS and lysed in 400 μl 50 mM Tris-HCl, 150 mM NaCl, 1% Triton X-100, protease inhibitor cocktail (Halt), pH 7.4 supplemented with either 2 mM CaCl_2_ or 2 mM EDTA, to identify whether proteins interact with Ca^2+^-bound CaM or Ca^2+^-free (Apo-) CaM respectively. Cleared lysates were then incubated with 30 μl Calmodulin beads (GE Healthcare) for 2 hrs or O/N, rotating end over end at 4°C. Beads were spun down and washed in Lysis buffer 6 times. Elution was carried out in Lysis buffer supplemented in either 10 mM EDTA or 10 mM CaCl_2_ (at the opposite condition used for lysis) at RT for 30 mins with gentle shaking. In these conditions, a protein that interacts with Apo-CaM binds in the presence of EDTA and is released by the addition of CaCl_2_ and *vice versa*. Elutes were then resuspended in 4X sample buffer. Lysates and pulldown fractions were run on SDS-PAGE as described above the presence of GFP-tagged proteins in the elutes was analyzed with an anti-GFP antibody (see key resources table).

#### Yeast Two-Hybrid Analysis

Yeast two-hybrid screening was performed by Hybrigenics Services, S.A.S., Evry, France (http://www.hybrigenics-services.com).

The coding sequences for *S. flexneri* OspC1 (aa 1-477), OspC2 and OspC3 (aa 1-484) were PCR-amplified and cloned into pB27 as a C-terminal fusion to LexA (LexA-OspC1-3). The construct was checked by sequencing the entire insert and used as a bait to screen a cDNA library of human macrophages stimulated by Pam3CSK4 or IFNβ constructed into pP6. pB27 and pP6 derive from the original pBTM116 (Vojtek and Hollenberg, 1995) and pGADGH (Bartel et al, 1993) plasmids, respectively.

In the case of OspC3, 65 million clones (5-fold the complexity of the library) were screened using a mating approach with YHGX13 (Y187 ade2-101::loxP-kanMX-loxP, matα) and L40ΔGal4 (mata) yeast strains as previously described (Fromont-Racine et al., 1997). 280 His+ colonies were selected on a medium lacking tryptophan, leucine and histidine. The prey fragments of the positive clones were amplified by PCR and sequenced at their 5’ and 3’ junctions. The resulting sequences were used to identify the corresponding interacting proteins in the GenBank database (NCBI) using a fully automated procedure. A confidence Predicted Biological Score was attributed to each interaction as previously described (Formstecher et al., 2005).

#### Organ CFU, RNA and protein extractions

Post-mortem, ceca and colons were harvested, flushed with cold PBS and cut longitudinally in 3 pieces. One piece was placed in gentamicin (400 μg/mL) for 30 mins, washed 5x and homogenized in 1% Triton X-100 for enumeration of CFU. The other sections were either homogenized in 1mL Trizol (Invitrogen cat. 15596026) as above for RNA extraction or fixed for histology purposes. Post-mortem spleens were homogenized in 1% Triton X-100 for enumeration of CFU. For CFU determination, serial dilutions were made in PBS and plated on TSB plates containing 0.01% CR and 100 mg/mL streptomycin. RNA extraction was carried out following Invitrogen user guide. For protein extraction, cells were lysed in 1%Triton X-100 and protein concentration were determined by Bradford assay. Colon length was calculating using Fiji software.

#### Histology

Day 1 post infection, colons were isolated, flushed, cut longitudinally, fixed in 10% neutral formalin for 24 hrs prior to be transferred in 70% ethanol. Samples were processed by the Crick histology platform following routine histologic methods. In brief, tissues were embedded in paraffin, sectioned at 3-5 μm thickness on a rotary microtome, and mounted on glass slides. Sections were stained with hematoxylin and eosin and coverslipped. Histopathological scoring of the organs was performed by a board-certified veterinary pathologist (SLP) who was blinded to the experimental groups at the time of the evaluation.

#### Cell death and viability assays

Cell death was quantified by measuring LDH release to the supernatant. To measure LDH, LDH cytotoxicity detection kit (Thermo Scientific) was used, according to the manufacturer’s instructions. To normalize for spontaneous cell lysis, the percentage of cell death was calculated as follows: (LDHsample – LDHnegative control)/ (LDHpositive control – LDHnegative control) × 100. Cell death was also analyzed by the uptake of propidium iodide (PI). Briefly, prior to infection, cells were incubated for 30 minutes with complete medium supplemented with 5 μg/mL PI (Thermo Scientific). Fluorescence was measured at 630 nm every hour with a Polarstar Omega plate reader at the indicated time points. Data were expressed as the % of fluorescence signal from cells lysed with a final concentration of 0.05% Triton-X100. Lastly, cell viability was determined using the CellTiterGlo luminescence detection assay (Promega) which quantifies intracellular ATP levels, according to the manufacturer’s instructions.

#### Bioinformatics analyses

To identify OspC homologs in other bacterial species, the *Shigella flexneri* OspC3 amino acid sequence was subject to BLASTp (Altschul et al., 1990) analysis against the entire NCBI database excluding the *Shigella* taxid using default parameters. FASTA files for the top 100 most similar proteins were extracted, and aligned with Clustal Omega (Sievers and Higgins, 2018). The sequence alignment was used to generate a phylogenetic tree in Interactive Tree of Life (iTol) (Letunic and Bork, 2021) using the maximum likelihood algorithm. For presentation, the tree was pruned to contain only a single sequence per genus in each individual clade of the tree. Amino acid sequences of the identified proteins were further analyzed with PREFFECTOR (Dhroso et al., 2018) to predict their propensity to be T3SS effectors. For prediction of potential secondary structures, amino acid sequences were queried using PSIPRED (Buchan and Jones, 2019; McGuffin et al., 2000). The resulting.PDB file was used to annotate sequence alignments using ESPript (Gouet et al., 2003).

The atomic model of OspC1 was generated with AlphaFold (Jumper et al., 2021), with default parameters. The structure of the OspC1-CaM complex was modelled using AlphaFold-Multimer. For this, an initial model was generated using the full-length protein sequences of both OspC1 and CaM. This led to a set of models where CaM was consistently binding around helix 1; however, for some of them, the confidence score was low. Upon inspection of the models, it became apparent that this was because in some of them, either the intrinsically-disordered N-terminus, or the C-terminal domain, was clashing with the putative position of CaM. We therefore performed a second modelling experiment, using only the N-terminal domain of OspC1. This led to a set of highly-similar, high-confidence models of the complex, supporting the reported binding mode. All structural figures were generated in ChimeraX.

### Quantification and statistical analysis

Data are shown as the means ± SEM. All statistical comparisons were performed using Prism 8 or 9 (GraphPad). Figure legends indicate the specific statistical tests used for each experiment. Statistical significance was considered as P<0.05.

## Data Availability

According to Wellcome Trust’s Policy on data, software and materials management and sharing, sequencing data are freely available in GEO under accession code GSE200447. Any additional data of this study are available from the lead contact upon request.
